# Antimicrobial Use Indices—The Value of Reporting Antimicrobial Use in Multiple Ways Using Data From Canadian Broiler Chicken and Turkey Farms

**DOI:** 10.3389/fvets.2020.567872

**Published:** 2020-10-19

**Authors:** Agnes Agunos, Sheryl P. Gow, David F. Léger, Anne E. Deckert, Carolee A. Carson, Angelina L. Bosman, Stefanie Kadykalo, Richard J. Reid-Smith

**Affiliations:** Public Health Agency of Canada, Center for Foodborne, Environmental and Zoonotic Infectious Diseases, Guelph, ON, Canada

**Keywords:** antimicrobial use, indicators, turkeys, broiler chickens, surveillance, Canada

## Abstract

We have previously described the importance of using multiple indicators for reporting national farm-level antimicrobial use (AMU) information, but the distribution of flock-level AMU and how these indicators relate to each other has not yet been fully explored. Using farm-level surveillance data (2013–2019), for broiler chickens (*n* = 947 flocks) and turkeys (*n* = 427), this study aims to (1) characterize flock-level AMU and identify high users, (2) identify appropriate AMU indicators and biomass denominator [population correction unit (PCU) vs. kg weight at pre-slaughter], and (3) make recommendations on the application to veterinarian-producer and national-level reporting. Diverse AMU patterns were identified in broiler chickens (156 patterns) and turkeys (68 patterns); of these, bacitracin, reported by 25% of broiler chicken and 19% of turkey producers, was the most frequently occurring pattern. Depending on the indicator chosen, variations in reported quantity of use, temporal trends and relative ranking of the antimicrobials changed. Quantitative AMU analysis yielded the following results for broiler chickens: mean 134 mg/PCU; 507 number (*n*) of Canadian (CA) defined daily doses (DDDvet) per 1,000 chicken-days and 18 nDDDvetCA/PCU. Analysis in turkey flocks yielded the following: mean 64 mg/PCU, 99 nDDDvetCA/1,000 turkey-days at risk and 9 nDDDvetCA/PCU. Flocks were categorized based on the percentiles of the mg/PCU distribution: “medium” to “low” users (≤75th percentile) and “high” users (>75th percentile). The odds of being a high user in both broiler chickens and turkeys were significantly increased: if water medications were used, and if trimethoprim-sulfonamides, bacitracins, and tetracyclines were used. Pairwise correlation analysis showed moderate correlation between mg/PCU and the nDDDvetCA/1,000 animal days at risk and between mg/PCU and nDDDvetCA/PCU. Significantly high correlation between nDDDvetCA/1,000 animal days at risk and nDDDvetCA/PCU was observed, suggestive that either of these could be used for routine monitoring of trends in AMU. One source of discrepancy between the indicators was the antimicrobial. Understanding the choice of parameter input and effects on reporting trends in AMU will inform surveillance reporting best practices to help industry understand the impacts of their AMU reduction strategies and to best communicate the information to veterinarians, their producers, and other stakeholders.

## Introduction

In recent years, methodologies for monitoring antimicrobials intended for use in animals have advanced and improved, which complements national and global priorities for mitigating the impact of antimicrobial resistance (AMR) using a One Health approach (1). The OIE has provided guidelines for data collection and reporting (2, 3) and published its 4th annual report with the global data on quantities of antimicrobials intended for use in animals expressed in milligrams per kilogram of animal biomass (mg/kg) (2). Recently (August 2019), the European Surveillance for Veterinary Antimicrobial Consumption (ESVAC) project under the European Medicines Agency has also published the reporting requirements for veterinary medicinal products (VMP) used in animals (4). The ESVAC implementing measures cite the use of milligrams of active substance adjusted by species population correction unit (mg/PCU), number of Defined Daily Doses (DDDvet) adjusted by species PCU (DDDvet/PCU) and Defined Course Dose for animals adjusted by species PCU (DCDvet/PCU) for reporting (4).

The use of multiple antimicrobial use (AMU) metrics (technical units of measurements such as frequency of use, number of medicated rations, days medicated, milligrams, number of DDDs) and indicators (an AMU metric in relation to a denominator such as the animal biomass and animal-time units) for AMU surveillance reporting has been previously described (5, 6). Multiple metrics and indicators are routinely used by the Canadian Integrated Program for Antimicrobial Resistance Surveillance (CIPARS) to better understand the temporal shifts in AMU and AMR in relation to poultry industry-wide AMU reduction strategies (7). The use of multiple AMU indicators is valuable, as interpretation of the surveillance data is highly influenced depending on the indicator chosen, thus multiple indicators provide a more complete picture of AMU. In the context of poultry production, when making comparisons across AMU indicators, there are several factors which can influence the resulting estimates, such as dose of the antimicrobial active ingredient (AAI), mortality levels (suggestive of a disease condition) and timing of administration of the antimicrobial (8). A change in an input parameter, such as the contextualizing denominator (animal biomass), can thus impact the overall interpretation of the AMU findings (9).

In Canada, recent changes in AMU regulations require a veterinary prescription and a valid veterinarian-client-patient relationship for the administration of medically important antimicrobials in animals (10). Recommended levels of drug or feed inclusion rates are indicated in the Compendium of Medicating Ingredients Brochure (11) and Compendium of Veterinary Products (12). All medically-important antimicrobials according to Health Canada's Veterinary Drugs Directorate's List A: List of certain antimicrobial active pharmaceutical ingredients (13) requires veterinary prescription.

In Canada, broiler chickens and turkeys are sold under a quota system, through which they are supply managed by national and provincial marketing boards. Food-borne pathogens resistant to antimicrobials deemed as high priority critically-important antimicrobials such as the 3rd generation cephalosporins and fluoroquinolones (14) and isolates with multiclass resistance are routinely detected from poultry products in Canada (7). Because of the food safety implications of these organisms, the larger poultry industry and allied industries (feed sector) developed “Responsible antimicrobial use in the Canadian chicken and turkey sector” guidelines in 2016 (15). Building on this strategy, sector-specific AMU policies were implemented to progressively eliminate the preventive use of medically important antimicrobials. The Chicken Farmers of Canada's AMU policy aimed to eliminate the preventive use of certain antimicrobial classes: 3rd generation cephalosporins and fluoroquinolones (Step 1—May 2014); aminoglycosides, macrolides, lincosamides, penicillins, trimethoprim and sulfonamides, and streptogramins (Step 2—end of 2018), and; bacitracins (Step 3—contingent upon reassessment of the impact of Step 2 by the end of 2020 (16). The turkey sector has also implemented a similar strategy) (17).

The Public Health Agency of Canada (PHAC) operates the Canadian Integrated Program for Antimicrobial Resistance Surveillance (CIPARS). CIPARS, which has been collecting AMU at the farm-level since 2013 in broiler chickens and turkeys. In the turkey sector, farm surveillance was initially implemented in one Canadian province (British Columbia) and progressively expanded to other provinces in collaboration with the establishment of FoodNet Canada (FNC) sentinel sites. FNC is another surveillance program also within PHAC, with a food safety and One Health theme. We have previously highlighted the early indications of the impact of Step 1 of the broiler chicken sector's AMU strategy (18) and the value of using multiple AMU indicators and integration of data to track the impact of this strategy (19). It is envisaged that the CIPARS farm component will enable informed decision-making by the industry, veterinarians and producers, in order to optimize AMU stewardship and preserve the effectiveness of antimicrobials currently available for use in the poultry sector in Canada. Using flock-level data collected across Canada (2013–2019), this study aimed to: (1) identify high users of antimicrobials using routine AMU analysis, (2) identify appropriate AMU indicators for reporting and compare AMU indicators with biomass denominators derived from two time points, average weight at treatment (PCU) vs. average weight at pre-slaughter (kg), and (3) make recommendations on the application to national-level data. The results will inform surveillance reporting best practices to help industry understand the impacts of their AMU reduction strategies and to best communicate this information to veterinarians and their producers.

## Materials and Methods

### Data Source

Farm data, collected by broiler chicken and turkey producers via their veterinarians using CIPARS species-specific questionnaires (Supplementary Material 1), between 2013 and 2019 were entered into the CIPARS AMU PostGresSQL database and extracted into Microsoft Excel (Office). Detailed farm-level methodology has been previously described (7, 19). Although other farm-level data on management and flock characteristics were available, for the purposes of this analysis we utilized the basic farm characteristics data such as flock inventory (birds at risk), pre-harvest live weight (defined as pre-slaughter weight in this paper; the weight ~1 week before shipment to slaughter plants), age of the birds at pre-harvest sampling (pertains to days at risk of being treated; from chick placement to pre-harvest sampling), stocking density in birds per square meter of floor space, the province and region where the flocks were raised and the frequency and quantity of antimicrobials used by all routes of administration. As previously, described (7, 19) a flock is defined as a group of birds originating from the same hatchery and placed approximately the same day in the sampling unit (e.g., barn, floor or pen).

### Data Analysis

All analyses were performed using Stata SE 15 (College Station, Texas).

#### Descriptive Statistics, Quantitative AMU Estimation, and Identification of High Users of AMU for Farm-level Reporting

##### Flock characteristics and frequency and quantity of AMU

Farm and flock-level characteristics which included conventional, antibiotic-free (ABF), raised without antibiotics (RWA), organic and other flock classifications were descriptively summarized. Ten broiler chicken flocks with partial or missing data were excluded from the analysis. Further analysis (pairwise correlation, comparison and identification of high users) included only the conventional flocks, regardless of their AMU exposures during the grow-out period. Excluded were flocks raised as ABF, RWA, and organic intended for the mainstream market because the decision not to use antimicrobials was market-driven and not based on flock health or production efficiency goals.

The first step involved routine CIPARS AMU data summaries and analyses (Table 1) and such as frequency of use by route of administration (number of flocks treated divided by the total flocks surveyed), patterns of AMU, duration of treatment, weight at treatment, days exposed, and level of drug/inclusion rates. For the purposes of our analysis, a flock AMU pattern was determined by combining all the AAI exposures via any route of administration during the life span of the bird. Flock-level estimates of antimicrobials administered via feed, water and injection were calculated using equations based on previously described methodology (7, 19). Briefly, within the CIPARS AMU database, the AAIs administered via feed (e.g., pre-starter, starter, grower, finisher, roaster or developer rations) were calculated using simple regression and integral calculus based on ration information (age at start of the ration and days the ration was fed) and using feed consumption charts for the breeds commonly used in Canada to obtain feed consumption for each ration per bird. This amount was then multiplied by the number of birds exposed, converted to tons and then multiplied by the reported level of drug per AAI. The level of drug pertains to the AAI inclusion rates in grams per ton of feed (11). For AAI administered via the drinking water, the quantity of use was estimated either by: (1) mg AAI per liter of drinking water multiplied by the estimated (calculated as described above for feed) water consumed during the course of treatment, or, (2) the total number of VMPs used during the course of treatment multiplied by the concentration of the AAI/s. For AAIs administered at the hatchery via *in ovo* or subcutaneous injections, the mg per hatching egg/broiler chick (or poult) was multiplied by the total number of broiler chicks or poults placed in the sampled barn. All values were converted to mg AAI for further AMU quantification.

**Table 1:** Methods used to calculate antimicrobial use for surveillance data collected from sentinel broiler chicken and turkey farms, 2013–2019.

**Measurements**	**Numerator**	**Denominator**
1. Frequency	No. of flocks using antimicrobials	Total no. of flocks surveyed
2. Days exposed per AAI	No. of days exposed	N/A
3. mg AAI	*Feed:* Ton fed × level of drug in the feed in grams × 1,000 *Water:* g AAI per liter of water × 1,000 *Injections:* mg AAI injected per bird or hatching egg	
**Routine CIPARS AMU estimation methodology**		
4. ${\text{mg/PCU}}_{\text{Br}}^{\text{a}}$ and ${\text{mg/PCU}}_{\text{Tk}}^{\text{a}}$	By class: mg of all AAIs (#3) Total flock: mg of all classes	Broilers: Birds at risk × 1 kg ESVAC average weight at treatment Turkeys: Birds at risk × 6.5 kg ESVAC average weight at treatment
5. nDDDvetCA/1,000 broiler chicken-days at risk^a^ and nDDDvetCA/1,000 turkey-days at risk^a^	mg adjusted by the DDDvetCA standard By class: nDDDvetCA^c^'s of all AAIs Total flock: nDDDvetCA's of all classes	Broilers: Birds at risk × 1 kg ESVAC average weight at treatment × days at risk Turkeys: Birds at risk × 6.5 kg ESVAC average weight at treatment × days at risk
6. ${\text{nDDDvetCA/PCU}}_{\text{Br}}^{\text{a}}$ and ${\text{nDDDvetCA/PCU}}_{\text{Tk}}^{\text{a}}$	As above in #5	As above in #4
**Alternate AMU estimation methodology**		
7. ${\text{mg/kg}}_{\text{Br}}^{\text{b}}$ and ${\text{mg/kg}}_{\text{Tk}}^{\text{b}}$	By class: mg of all AAIs, all routes (as in #4) Total flock: mg of all classes, as in #4	Broilers: Birds at risk × kg broiler biomass^1^ Turkeys: Birds at risk × kg turkey biomass^1^
8. nDDDvetCA/1,000 broiler chicken days at risk^b^ and nDDDvetCA/1,000 turkey-days at risk^b^	By class: nDDDvetCAs of all AAIs (as in #5) Total flock: nDDDvetCA's of all classes (as in #5)	Broilers: Birds at risk × kg broiler biomass × days at risk Turkeys: Birds at risk × kg turkey biomass × days at risk
9. ${\text{nDDDvetCA/kg}}_{\text{Br}}^{\text{b}}$ and ${\text{nDDDveCA/kg}}_{\text{Tk}}^{\text{b}}$	As above in #8	(As in #7)

1 *Broiler chicken and turkey average pre-slaughter live weights in kg (the animal biomass)*.

a *Based on routine CIPARS formula (7, 19)*.

b *kg broiler chicken and turkey live pre-slaughter weights*.

c *DDDvetCA—defined daily doses for animals using Canadian standards. DDDvetCA standards described elsewhere (20)*.

*AAI, antimicrobial active ingredient*.

*nDDDvetCA, number of defined daily doses for animals using Canadian standards*.

*ESVAC, European Surveillance for Veterinary Antimicrobial Consumption*.

*PCU, population correction unit*.

*N/A, not applicable*.

*Br, broilers*.

*Tk, turkeys*.

The second step categorized flocks based on the percentiles of the resulting mg/PCU distribution as “medium” to “low” users (≤75th percentile) and “high” users (>75th percentile). Differences between high and medium- to-low users were examined more closely using logistic regression and exact logistic regression where appropriate. Independent variables investigated included route of administration and antimicrobial class used. Milligrams per PCU differed significantly between provinces for chickens and between turkey weight categories for turkeys and therefore these variables were included as fixed effects in the respective analyses. Due to the industry AMU reduction strategy that was implemented during the period of this study, year was also forced into the analyses.

#### Comparison of AMU Indicators and Exploration of Alternate Weights Used in the Denominators

##### Pairwise Comparisons of AMU Indicators

The purpose of pairwise comparisons of indicators was to inform the selection of the most appropriate indicator (s) for communicating AMU surveillance results to the poultry industry and for providing feedback to veterinarians and their producers. The three different flock-level AMU indicators were assessed for correlation using Pearson's correlation coefficient (PCC). A *P-*value of 0.0001 was considered significant for each of the correlation pairs shown in **Tables 6**, **7** and Supplementary Material 2.

##### Exploration of alternate weights in the denominators

The purpose was to explore the impact of different choices of weights of the animals on the reported AMU indicator. For these analyses, the same equations were used but the actual recorded pre-slaughter live weight was applied to the denominator, replacing the PCU or average weight at treatment of 1 kg and 6.5 kg for broiler chickens and turkeys, respectively. In brief, flock-level AMU was estimated based on numerator and denominator input parameters described in Table 1. The Canadian defined daily doses for animals (DDDvetCA) standards developed for broiler chickens and turkeys (20) were used to estimate the total number (*n*) of defined daily doses in animals using Canadian standards (nDDDvetCA). Route-specific DDDvetCA standards were applied. The pre-slaughter weight was used as a surrogate for slaughter weight (actual weight at slaughter) and actual days at risk (as per routine CIPARS analysis) were used. For the dose-based indicator, nDDDvetCA/1,000 broiler chicken (or turkey)-days at risk, or the proportion of animals treated daily with an average dose, was based on previously described methodologies (21, 22).

#### Overall National AMU Estimation and Temporal Trends

This section aimed to update the previous AMU results reported by CIPARS for national farm-level data, applying routine methods and those used above (exploration of alternate weights in the denominators) (7, 23). National data were estimated as previously described using the sum of milligrams of AAI used, total nDDDvetCA's and the total bird population at risk. The national estimates using both routine and the alternative weight (pre-slaughter weight) AMU indicator were plotted in Microsoft Excel (Office).

## Results

### Broiler Chickens

#### General Description of AMU and Flock/Farm Characteristics

##### Flock and farm characteristics

A cumulative total of 934 broiler chicken flocks across 5 Canadian provinces participated in the CIPARS broiler chicken farm surveillance between 2013 and 2019 (British Columbia: 204 flocks, Alberta: 195 flocks, Saskatchewan: 59 flocks, Ontario: 279 flocks and Québec: 197 flocks). Overall, the flocks sampled during the 7 years encompassed 22 million birds (~3 million birds/year) or the equivalent of 48 million kg of broiler chicken biomass (~6 million kg/year). Descriptive statistics for flock-level characteristics are summarized in Table 2.

**Table 2:** Characteristics of the studied broiler chicken flocks (*n* = 934), 2013–2019.

**Characteristics**	**Units**	**Mean of flocks (standard error of the mean)**
Age sampled/days at risk	Days	35 (0.1)
Pre-slaughter live weights^a^	kg	2 (0.01)
Birds at risk	n birds	23,735 (441)
Total pre-slaughter live weight	kg, total	47,873 (916)
Stocking density	birds/m^2^	11 (0.01)
Farm capacity	*n* birds	62, 311 (1,936)
Mortality	%	4 (0.1)

a *Used interchangeably with pre-harvest throughout the manuscript (farm visit and data collection before shipment for slaughter)*.

##### Antimicrobial active ingredients and routes of administration

There were 23 AAIs used in broiler chickens (Table 3). Seven AAIs were administered via feed (tylosin, procaine penicillin, virginiamycin, trimethoprim and sulfadiazine, bacitracin, oxytetracycline, and avilamycin). Thirteen AAIs were administered via water (enrofloxacin, apramycin, amoxicillin, lincomycin, penicillin, penicillin and streptomycin, oxytetracycline and neomycin, tetracycline, tetracycline and neomycin, sulfadimethoxine, sulfaquinoxaline, and sulfaquinoxaline and pyrimethamine). Three AAIs were administered via subcutaneous and *in ovo* injections (ceftiofur, gentamicin and lincomycin and spectinomycin).

**Table 3:** Reported antimicrobial use by route of administration by antimicrobial active ingredient in broiler chicken flocks, 2013–2019.

							**Indicators mean (standard error of the mean)**		
	***n* (%) flocks treated^**a**^**	**Total birds treated (‘000)**	**Days exposed, mean (min-max)**	**No. (%) of treatments^**b**^**	**kg weight at treatment, mean (min–max)**	**Level of drug or inclusion rates, mean (min–max)**	**mg/PCU**	**nDDDvetCA/1,000 broiler-chicken days**	**nDDDvet/PCU_**Br**_**
**Injection (*****in ovo*** **or subcutaneous)**				**No. (%) injections**		**ml/chick**			
Ceftiofur	39 (4%)	1,022	1	39 (1%)	0.04	0.1	0.1 (0.01)	1 (0.1)	0.05 (0.004)
Gentamicin	36 (4%)	867	1	37 (1%)	0.04	0.2	0.2 (0.01)	1 (<0.01)	0.02 (<0.01)
Lincomycin-spectinomycin	177 (19%)	4,014	1	177 (5%)	0.04	0.75	1 (0.02)	4 (0.1)	0.13 (<0.01)
**Feed**				**No. (%) medicated rations**		**Grams/ton**			
Avilamycin	213 (23%)	5,160	18 (17–19)	434 (15%)	0.87 (0.07–2.32)	15 (15–30)	31 (1)	307 (12)	11 (0.43)
Bacitracin	509 (54%)	12,564	26 (25–27)	1,482 (52%)	0.95 (0.05–3.35)	55 (11–110)	144 (3)	406 (8)	14 (0.32)
Oxytetracycline	7 (1%)	143	10 (7–13)	7 (0.2%)	0.87 (0.41–1.22)	440 (97–440)	448 (106)	656 (131)	27 (6.34)
Penicillin procaine	83 (9%)	2,370	16 (15–17)	150 (5%)	0.61 (0.11–1.65)	55 (20–110)	56 (3)	318 (18)	10 (0.60)
Trimethoprim-sulfadiazine	81 (9%)	1,995	7 (6–8)	84 (3%)	0.93 (0.11–2.44)	300 (200–300)	175 (13)	768 (52)	27 (2)
Tylosin	91 (10%)	2,392	21 (19–23)	232 (8%)	0.82 (0.07–2.46)	22 (22–44)	43 (3)	48 (3)	2 (0.1)
Virginiamycin	192 (21%)	4,557	22 (21–24)	487 (17%)	0.99 (0.06–3.28)	22 (11–44)	48 (2)	481 (20)	17 (0.8)
**Water**				**No. (%) water treatments**		**Total mg/bird**^**c**^			
Amoxicillin	15 (2%)	398	6 (5–6)	16 (0.5%)	0.85 (0.11–1.59)	53 (14–443)	63 (14)	158 (37)	5 (1.2)
Apramycin	1 (0.1%)	40	4	1 (0.03%)	0.12	30	30	34	1
Enrofloxacin	3 (0.2%)	79	5 (3–6)	3 (0.1%)	0.12 (0.09–0.13)	0.5 (0.3–0.5)	0.4 (0.1)	2 (0.2)	0.1 (0.01)
Lincomycin	1 (0.1%)	10	2	1 (0.03%)	1.34	63	63	502	17
Penicillin	29 (2%)	675	5 (5–6)	31 (1%)	0.88 (0.07–2.04)	153 (8–432)	166 (21)	114 (14)	4 (0.5)
Penicillin-streptomycin	13 (1%)	569	4 (5–6)	19 (0.6%)	0.21 (0.07–1.09)	13 (7–321)	41 (18)	150 (77)	5 (3)
Sulfamethazine	9 (1%)	293	4 (3–5)	9 (0.3%)	0.25 (0.11–0.74)	136 (34–311)	137 (28)	19 (4)	1 (0.1)
Sulfaquinoxaline	12 (1%)	358	3 (3–4)	11 (0.3%)	0.55 (0.12–1.86)	66 (13–208)	80 (15)	32 (6)	1 (0.2)
Sulfaquinoxaline (*pyr*)^d^	7 (1%)	207	3 (2–4)	7 (0.0%)	1.22 (0.09–1.86)	12 (4–39)	15 (4)	37 (9)	1 (0.4)
Oxytetracycline-neomycin	1 (0.1%)	19	4	1 (0.03%)	0.2	66	66	78	3
Tetracycline	3 (0.3%)	64	4 (3–6)	3 (0.1%)	0.68	19 (16–113)	49 (32)	63 (40)	2 (1)
Tetracycline-neomycin	8 (1%)	326	4 (4–5)	8 (0.2%)	0.25 (0.18–0.49)	44 (24–233)	73 (26)	102 (37)	3 (1)

a *Number of flocks treated/total flocks surveyed*.

b *Number of treatments/ total treatments from all routes of administration*.

c *The estimated total milligrams administered per bird during the course of water treatment. This was reported as grams per liter of drinking water (2013–2018) or total grams of active ingredient administered during the course of treatment per bird in the flock treated*.

d *This is in combination with pyrimethamine (a coccidiostat); only the sulfaquinoxaline component was included in the estimates*.

##### Frequency

The vast majority of the flocks (83%) were medicated via feed (i.e., represented the greatest route of AAI exposures), 27% of flocks were medicated via injections, and a small percentage were medicated via water (10%). More than half of the producers used one (34%) or two (30%) AAIs during the grow-out period by all routes combined; the remaining flocks used three (16%), four (5%), and 5 or more AAIs (<1%). One hundred and three flocks (11%, excluded in subsequent AMU analysis as these flocks did not contribute to multiple units of comparisons) were intended for the mainstream market including RWA, ABF and organic without any exposures to medically-important antimicrobials, ionophores, or chemical coccidiostats. There were 3,239 treatment frequencies recorded (252 injections, 2,875 feed, 110 water).

##### AMU patterns

The data indicated that there were diverse AMU patterns (156 patterns) utilized by the broiler chicken producers (Supplementary Material 3). The most frequently occurring patterns were treatment of the flock with bacitracin (25%, *n* = 197 flocks), avilamycin-bacitracin (6%, *n* = 50 flocks), bacitracin-lincomycin and spectinomycin combination (5%, *n* = 42), avilamycin (5%, *n* = 38), and virginiamycin (5%, *n* = 36) during the broiler growing period. The diversity of AMU patterns decreased over time from 54 AMU patterns (highest in 2014) to 19 AMU patterns (2019).

##### Total birds exposed to AAIs

More than half of the total population sampled were medicated with bacitracin (57%, 12.6 million birds). Other notable bird exposures were avilamycin (23%, 5.2 million birds) and virginiamycin (21%, 4.6 million birds).

##### Duration of exposure to AAIs

In treated flocks, the mean number of medicated rations was 4 rations per flock and the mean days medicated was 30 days. Therefore conventional flocks, on average, were commonly exposed to medicated feed ~86% of the time during the growing period. For days of exposure to specific in-feed AAI's, the longest days of exposure were for bacitracin (mean: 26 days, range 25–27), followed by tylosin (mean 21 days, 19–23), and virginiamycin (mean 22 days, 21–24). The AAIs reportedly used for treatment, oxytetracycline and trimethoprim-sulfadiazine, had a mean of 10 and 7 days, respectively. For water administered AAIs, largely intended for treatment, the mean duration of treatment was relatively shorter and varied by AAIs from 2 days (lincomycin) to 6 days (amoxicillin) but the recommended duration of treatment (3–5 days) was used for the remaining AAIs (12). Injections were provided once at either day 18 of incubation (*in ovo*) or at day of hatch (subcutaneous) at the hatchery.

##### Age at treatment

**Figure 1:**
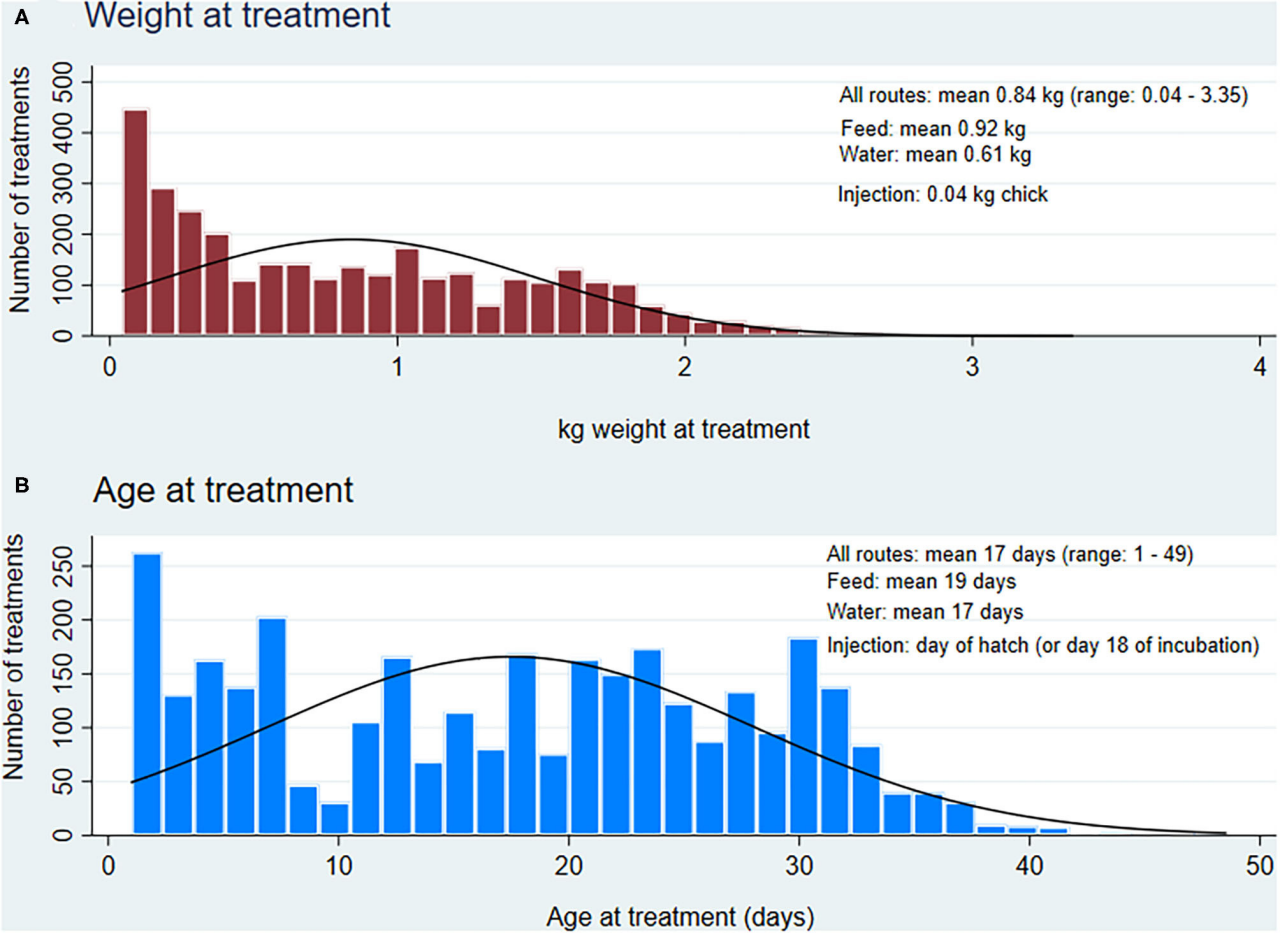
Distribution of weight and age at treatment (*n* = 3,876 treatments via feed, water and injection) for broiler chickens, 2013 to 2019. **(A)** Weight at treatment combines all the estimated weights for each treatment via injection, water and feed routes of administration, **(B)** Age at treatment combines all the reported age for each treatment via injection (default at day 1), water and feed routes of administration.

The mean age at treatment varied by route of administration and AAI (Figure 1, Table 3), but combined data from all routes yielded a mean of 17 days.

##### Weight at treatment

The mean weight at treatment also varied by route of administration and AAI (Figure 1, Table 3), but combined data from all routes yielded a mean of 0.84 kg, slightly lower than ESVAC's 1 kg.

##### Inclusion rates or level of drug

The quantity of AAIs (Table 3) administered via feed (grams/ton), water (g/liter of drinking water or mg/bird), and injection (mg/hatching egg or chick) were largely according to the approved claims indicated in the Compendium of Medicating Ingredients Brochure (11). For medicated rations, the inclusion rates of AAI in feed were consistent throughout the growing period (if used in multiple rations), except in cases where a stepwise approach for the drug administration with a changing inclusion rate during the growing period was used. For example, 110 g/ton of bacitracin in the pre-starter ration for “reduction of early mortality due to diminished feed consumption and chilling” was reduced to 55 g/ton in subsequent rations for the prevention of necrotic enteritis. Similarly, avilamycin was added at 15–30 g/ton as per product label and approved level of drug in the feed (11, 12).

##### Quantity of antimicrobials reported to be used

**Figure 2:**
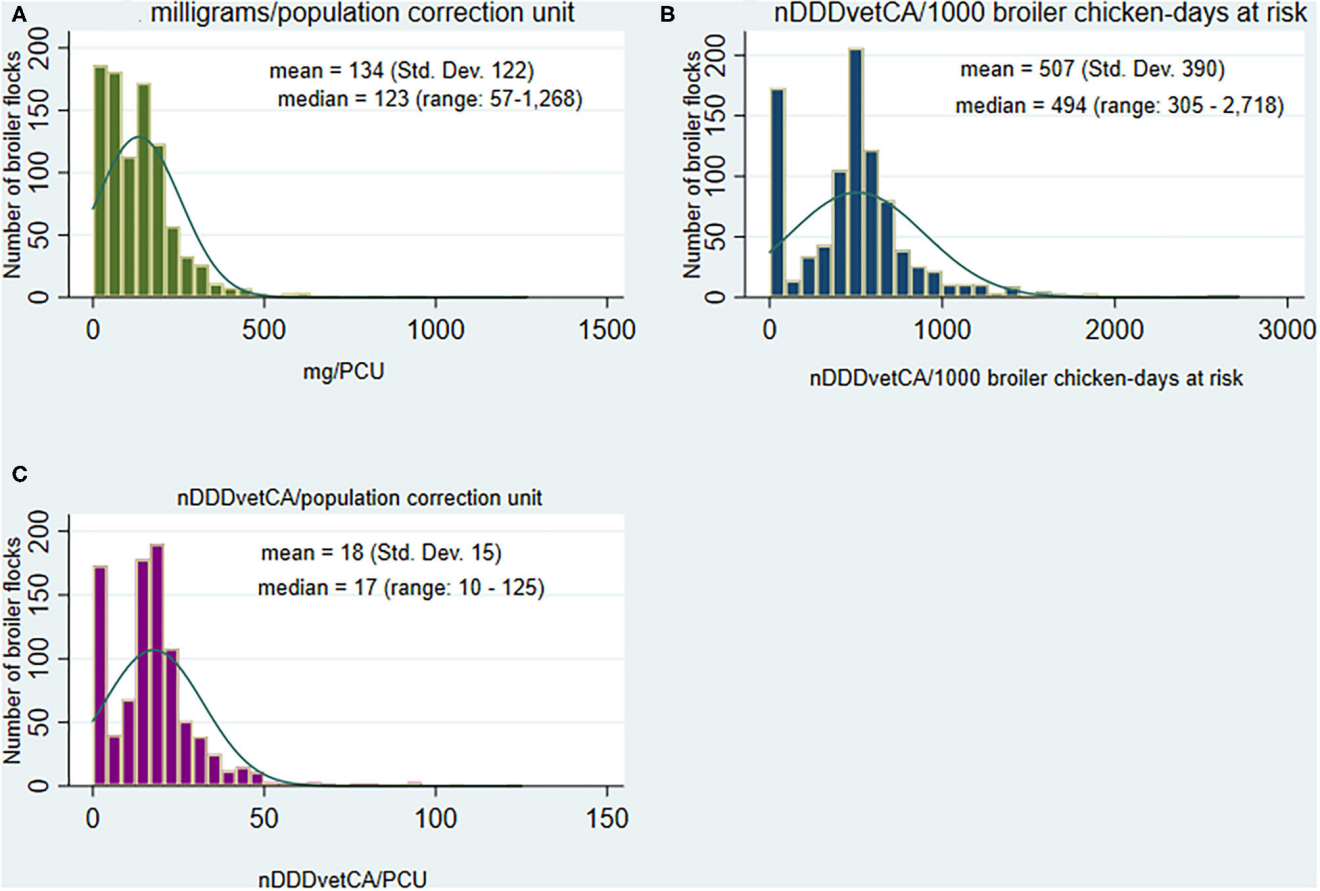
Distribution of the quantities of antimicrobials reported to be used, by antimicrobial use indicators, in broiler chicken flocks (*n* = 934 flocks), 2013 to 2019. **(A)** Broiler flock-level milligrams adjusted by population and weight (population correction unit), **(B)** Broiler flock-level number of defined daily doses for animals using Canadian standards adjusted by population, weight at treatment and days at risk, and **(C)**. Broiler flock-level number of defined daily doses for animals using Canadian standards adjusted by population and broiler weight (population correction unit).

The flock-level AMU data showed a skewed distribution where the mean values were higher than the median in the three AMU indicators; zeros represented flocks that were raised as ABF, RWA, organic or other production types not exposed to any AAI [(24); Figure 2]. Across the participating flocks, the mean AMU at the flock-level in mg/PCU_Br_ was 134 (median: 123; minimum: 57 and maximum: 1,268). When adjusted for dose and animal-time parameters, nDDDvetCA/1,000 broiler chicken-days at risk and nDDDvetCA/PCU_Br_, the mean was 507 (median: 494; 305–2, 713) and 18 (median: 17; 10–125), respectively (Figure 2).

Descriptive statistics by AAI for the weight-based and dose-based AMU indicators are shown in Table 3. Data aggregated by antimicrobial class and the distribution of the flock-level mg/PCU_Br_ are also presented in Supplementary Material 1. The three highest means in mg/PCU_Br_ were flocks that were medicated with the following classes: tetracyclines (*n* = 19 flocks, 190 mg/PCU_Br_), trimethoprim-sulfonamides (*n* = 106 flocks; 157 mg/PCU_Br_), and bacitracins (*n* = 509; 144 mg/PCU_Br_). For nDDDvetCA/1,000 broiler-chicken days at risk, the relative ranking of the antimicrobial classes changed and the highest means were flocks that consumed trimethoprim-sulfonamides, streptogramins, and bacitracins at 594, 481, and 406 nDDDvetCA/1,000 broiler chicken-days at risk, respectively. For nDDDvetCA/PCU, the highest means paralleled the previous indicator at 21, 17, and 14 nDDDvetCA/PCU for trimethoprim and sulfonamides, streptogramins, and bacitracins, respectively.

#### Identification of High Users of AMU in Broiler Chickens

Flocks defined as high users based on mg/PCU_Br_ were significantly (P ≤ 0.05) more likely to have used antimicrobials in water [Odds ratio (OR) = 6.49]. These were conventional flocks that were treated with antimicrobials via feed for routine necrotic enteritis prevention, plus medicated via water for treatment of diseases other than necrotic enteritis. For example, when diagnosed with any of the lesions associated with avian pathogenic *E. coli* (APEC) including yolksacculitis, septicemia or airsacculitis, and occasionally vertebral osteomyelitis (*Enterococcus cecorum*) or *Staphylococcus aureus* (osteomyelitis) infections. These flocks were also significantly more likely to have used aminoglycosides (OR = 3.41), bacitracins (OR = 4.27), penicillins (OR = 2.47), tetracyclines (OR = 9.17), and trimethoprim-sulfonamides (OR = 13.34). As well, these flocks were significantly (P ≤ 0.05) more likely to be in the top 25th percentile of aminoglycosides (OR = 3.42), bacitracins (OR = 74.48), and penicillins (OR = 5.95) users based on mg/PCU_Br_. Flocks using macrolides (OR = 0.36), streptogramins (OR = 0.41), and orthosomycins (OR = 0.43) were significantly (P ≤ 0.05) less likely to be classified as high users based on mg/PCU_Br_. It should be noted that all of the antimicrobial classes listed above were administered through multiple routes.

#### Turkeys

#### General Description of AMU and Flock/Farm Characteristics

##### Flock and farm characteristics

A cumulative total of 427 turkey flocks across four Canadian provinces participated in the CIPARS turkey farm surveillance between 2013 and 2019 (British Columbia: 206 flocks, Alberta: 20 flocks, Ontario: 121 flocks and Québec: 80 flocks). The total birds sampled were 3.2 million birds (~0.25–0.68 million birds/year) equivalent to 29 million kg turkey biomass (~2–6 million kg/year). Descriptive statistics for farm and flock characteristics by marketing weight categories are summarized in Table 4.

**Table 4:** Characteristics of the studied turkey flocks (*n* = 427) by marketing weight categories, 2013–2019.

		**Mean of flocks (Standard error of the mean)**					
**Characteristics**	**Units**	**Broiler turkeys** **(*n* = 84)**	**Light hens** **(*n* = 105)**	**Heavy hens** **(*n* = 67)**	**Light tom** **(*n* = 48)**	**Heavy tom** **(*n* = 123)**	**Overall** **(*n* = 427)**
Age sampled	Days	64 (1)	78 (1)	96 (1)	96 (1)	107 (1)	89 (1)
Preslaughter live weight^a^	kg bird	5 (0.1)	7 (0.1)	9 (0.2)	12 (0.2)	15 (0.2)	10 (0.2)
Birds at risk	*n* birds	8, 624 (478)	8,215 (453)	7,596 (562)	7,131 (464)	6,171 (279)	7,488 (198)
Total preslaughter live weights	kg, total	42, 457 (2,552)	55,122 (3,159)	68,277 (4,626)	87,546 (6,009)	89,773 (4,121)	68,321 (2,009)
Stocking density	birds/m^2^	6 (0.2)	6 (0.2)	5 (0.2)	3 (0.2)	3 (0.1)	5 (0.1)
Farm capacity	*n* birds	23, 704 (3, 844)	27,528 (2,190)	31,259 (2,865)	26,726 (3,061)	25,642 (2,095)	26,924 (1,242)
Mortality	%	4 (0.3)	5 (0.4)	6 (0.4)	6 (0.4)	8 (0.4)	6 (0.2)

a *Used interchangeably with pre-harvest throughout the manuscript (i.e., farm visit and data collection before shipment for slaughter)*.

The mean pre-harvest sampling age or days at risk across the turkey flocks sampled was 89 days and varied by marketing weight categories, and was shortest in broiler turkeys (64 days) and longest in heavy toms (107 days). Fifty-six percent of the producers raised birds in the heavier weight categories (combined heavy hens, light toms and heavy toms).

##### Antimicrobial active ingredients and routes of administration

There were 20 different AAIs used in turkeys (Table 5) and 8 AAIs were administered via feed (tylosin, procaine penicillin, virginiamycin, trimethoprim-sulfadiazine, bacitracin, chlortetracycline, oxytetracycline, and avilamycin), 10 in water (enrofloxacin, neomycin, amoxicillin, penicillin G potassium, oxytetracycline-neomycin, tetracycline-neomycin, sulfaquinoxaline, and sulfaquinoxaline-pyrimethamine), and 2 via injections (ceftiofur, gentamicin).

**Table 5:** Reported antimicrobial use by route of administration and by antimicrobial active ingredient in turkey flocks, 2013–2019.

							**Indicators, mean (standard error of the mean)**		
	***n* (%) flocks treated^**a**^**	**Total birds treated (‘000)**	**Days exposed, mean (min-max)**	**No (%) of treatments^**b**^**	**kg weight at treatment mean (min-max)**	**Level of drug mean (min-max)**	**mg/PCU_**Tk**_**	**nDDDvet/1,000 turkey-days at risk**	**nDDDvet/PCU_**Tk**_**
**Injection**				**No. (%) injections**		**mg/poult**			
Ceftiofur	1 (0.2%)	14	1	1 (0.1%)	0.06	0.2	0.03	<0.1	<0.1
Gentamicin	190 (44%)	1,563	1	190 (11%)	0.06	1	0.2	0.16	0.2
**Feed**				**No. (%) medicated rations**		**Grams/ton**			
Avilamycin	10 (2%)	74	40 (11–70)	24 (1%)	3.80 (0.26–11.76)	18 (15–25)	19 (5)	69 (13)	6 (2)
Bacitracin	181 (42%)	1,442	66 (6–110)	799 (46%)	3.08 (0.15–16.08)	55 (55–110)	96 (5)	103 (4)	9 (0.5)
Chlortetracycline	10 (2%)	102	16 (4–42)	12 (1%)	2.64 (0.26–6.83)	330 (220–440)	114 (30)	68 (15)	7 (2)
Oxytetracycline	2 (0.5%)	81	49	4 (0.2%)	1.36 (0.45–2.27)	440 (220–660)	182	99	11
Penicillin procaine	15 (4%)	4	26 (14–42)	25 (1%)	1.17 (0.26–3.02)	33 (33–110)	16 (3)	32 (6)	3 (0.5)
Trimethoprim-sulfadiazine	21 (5%)	139	13 (4–28)	22 (1%)	4.33 (0.55–11.76)	300 (200–300)	113 (22)	181 (32)	17 (3)
Tylosin	9 (2%)	61	59 (14–84)	35 (2%)	4.43 (0.26–11.76)	22 (22–22)	44 (10)	17 (4)	2 (0.4)
Virginiamycin	130 (30%)	1,054	64 (7–112)	548 (32%)	2.80 (0.26–13.95)	22 (16.5–44)	33 (2)	131 (5)	12 (1)
**Water**				**No. (%) water treatments**		**Total mg/bird**^**c**^			
Amoxicillin	5 (1%)	27	5 (4–6)	5 (0.3%)	2.99 (0.15–5.22)	63 (0.5–413)	20 (11)	17 (9)	2 (10)
Enrofloxacin	4 (1%)	40	4 (4–5)	4 (0.2%)	2.46 (0.37–6.16)	9 (5–13)	1 (0.27)	3 (1)	0.2 (0.1)
Neomycin	3 (1%)	25	5	3 (0.2%)	2.42 (1.97–3.31)	26 (25–401)	23 (19)	11 (9)	1 (1)
Penicillin	21 (5%)	156	7 (3–28)	23 (1.3%)	4.78 (0.26–13.95)	63 (4–1786)	38 (14)	10 (4)	1 (0.3)
Penicillin-streptomycin	7 (2%)	50	5 (1–8)	8 (0.5%)	1.36 (0.15–3.88)	4	1 (1)	2 (1)	1 (1)
Sulfaquinoxaline	2 (0.5%)	9	5 (3–6)	1 (0.1%)	5.07 (3.31–6.83)	85 (75–95)	13 (2)	2 (0.10)	0.2
Sulfaquinoxaline (pyr)^d^	1 (0.2%)	79	4	2 (0.1%)	4.37 (4.37–4.37)	12	2	2	0.2
Oxytetracycline-neomycin	1 (0.2%)	44	3	1 (0.1%)	4.37 (4.37–4.37)	55	9	4	9
Tetracycline	7 (2%)	7	6 (4–10)	5 (0.3%)	2.50 (0.52–4.98)	11 (0.03–227)	8 (5)	5 (3)	0.4 (0.2)
Tetracycline-neomycin	6 (1%)	3	9 (5–21)	9 (0.5%)	2.70 (0.15–11.75)	54 (24–186)	17 (5)	8 (3)	17 (5)

a *Number of flocks treated/total flocks surveyed*.

b *Number of treatments/total treatments from all routes of administration*.

c *The estimated total milligrams administered per bird during the course of water treatment. This was reported as grams per liter of drinking water (2013–2018) or total grams of active ingredient administered during the course of treatment per bird in the flock treated*.

d *This is in combination with pyrimethamine (a coccidiostat). Only the sulfaquinoxaline component was included in the estimates*.

##### Frequency

Most flocks (72%) were treated via the feed (i.e., represented the greatest route of AAI exposures as with broilers), nearly half of the flocks were medicated via injections (45%) and a small percentage were medicated via water (11%). Fifty-seven flocks (13%, excluded in subsequent AMU analysis as these flocks did not contribute to multiple units of comparisons) were intended for the mainstream market including RWA, ABF, and organic without any exposures to medically-important antimicrobials, ionophores, and chemical coccidiostats. As with the broiler chickens, more than half of the turkey producers used one (31%) or two (33%) AAIs during the grow-out period by all routes combined; the remaining flocks used three (10%), four (4%), and more than five AAIs (<1%). There were 1,721 total treatment frequencies recorded (191 injections, 1,469 feed, and 61 water). It is important to note that a single flock could have been exposed to AAIs via multiple routes of administration.

##### AMU patterns

Combined data from all routes indicated that there were different AMU patterns utilized by the turkey producers during the grow-out period, though less diverse compared to broilers (68 patterns). The most frequently occurring patterns were treatment of the flock with bacitracin (19%, *n* = 65), gentamicin-virginiamycin (17%, *n* = 57), bacitracin-gentamicin (15%, *n* = 50), virginiamycin (10%, *n* = 35), and gentamicin (28%, *n* = 268). Over time, the number of patterns decreased from 24 AMU patterns (2016, national program commenced) to 20 AMU patterns (2019) (Supplementary Material 3).

##### Total birds exposed to AAIs

Almost half of the total population sampled were medicated with gentamicin (49%, 1.6 million birds). Other notable antimicrobial exposures were bacitracin (35%, 1.4 million birds) and virginiamycin (33%, 1.05 million birds).

##### Duration of exposure to AAIs

In treated flocks, the mean number of medicated rations was 4 per flock (up to 8 rations in heavier weight categories). The mean exposure days in feed-administered AAIs were longest for bacitracin (66 days; 6–110), followed by virginiamycin (64; 7–112) and tylosin (59; 14–84). For AAIs administered via water, the mean days of exposure varied depending on the AAI but were relatively shorter than feed exposures from 3 days (oxytetracycline-neomycin) to 7 days (penicillin). The maximum durations for AAIs administered via water were documented for tetracycline-neomycin (21 days) and penicillin (28 days) reportedly used for the treatment of secondary bacterial infection and clostridial dermatitis, respectively.

##### Age at treatment

**Figure 3:**
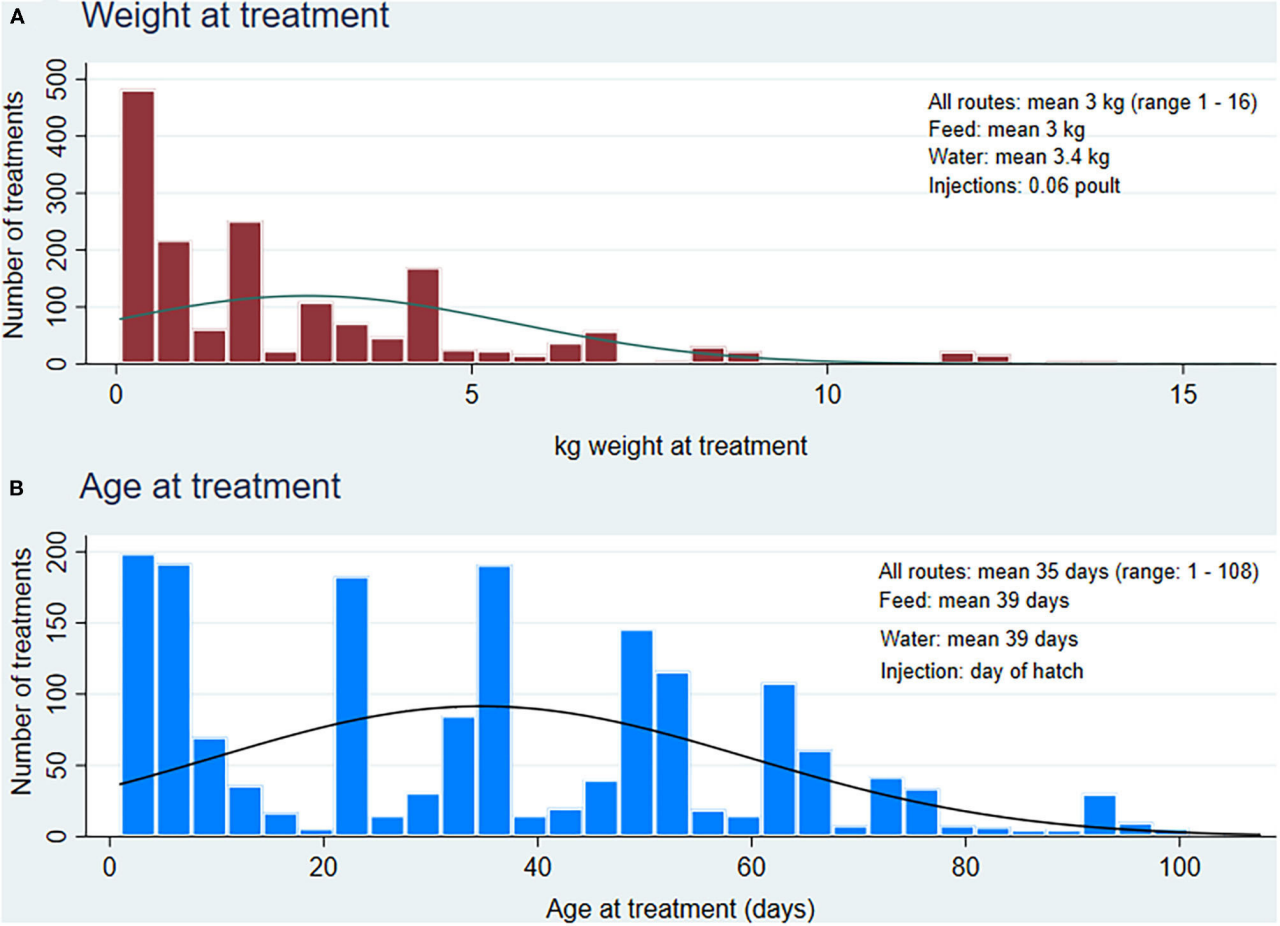
Distribution of weight and age at treatment in treated turkey flocks (*n* = 1,721 treatments via feed, water and injection), 2013 to 2019. **(A)** Weight at treatment combines all the estimated weights for each treatment via injection, water and feed routes of administration, **(B)** Age at treatment combines all the reported age for each treatment via injection (default at day 1), water and feed routes of administration.

The mean age at treatment varied by route of administration and AAI (Figure 3, Table 5), but combined data from all routes yielded a mean age of 35 days.

##### Weight at treatment

Similarly, the weight at treatment varied by route of administration and AAI, but combined data from all routes yielded a mean treatment weight of 3 kg (Figure 3, Table 5), relatively lower than ESVAC's 6.5 kg.

##### Inclusion rates or level of drug in feed, water, and injection

As with the broiler chickens, the amount of AAIs added through feed, drinking water and injections were largely according to the Compendium of Medicating Ingredients Brochure (11) and the Compendium of Veterinary Products (12).

##### Quantity of antimicrobial use reported

**Figure 4:**
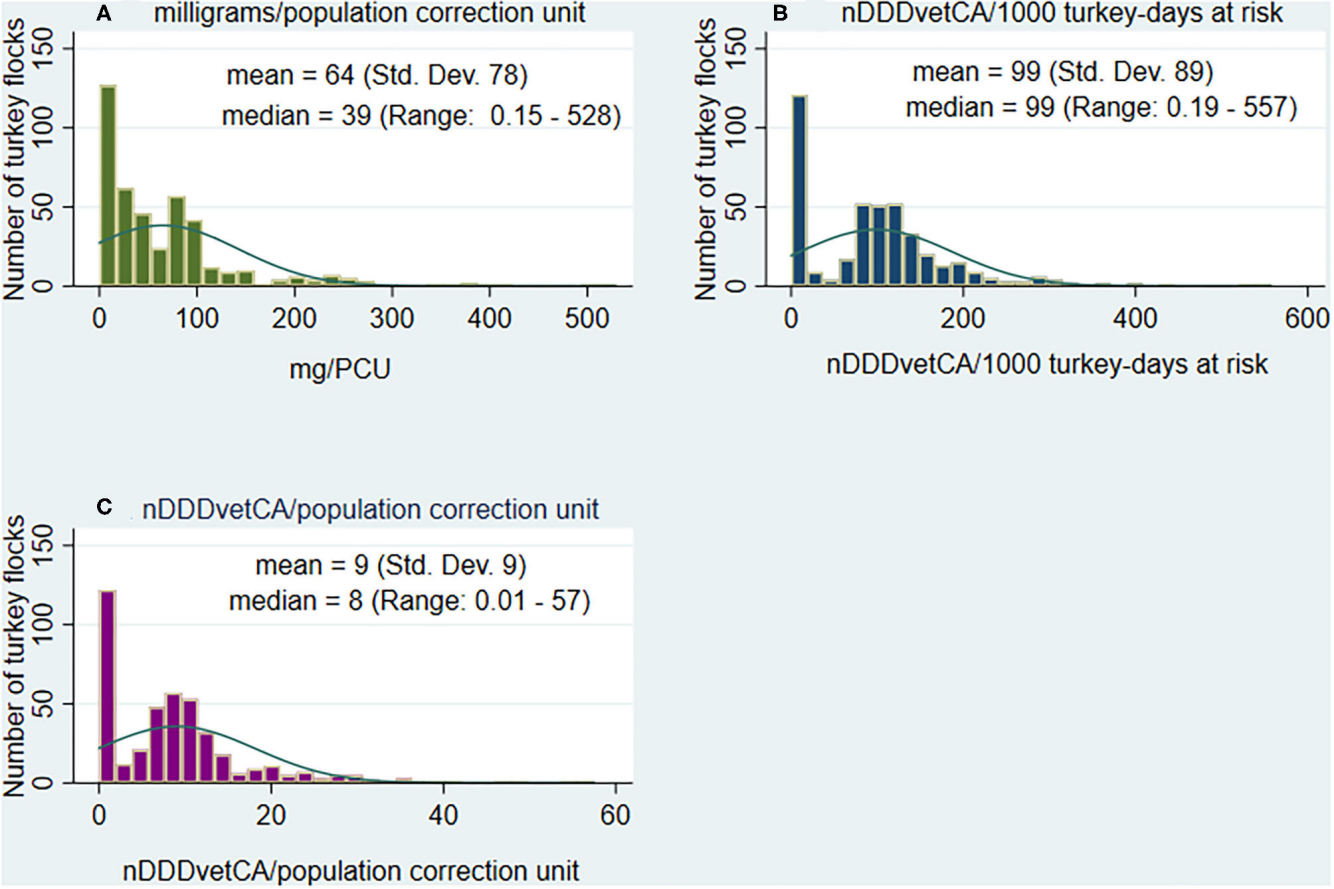
Distribution of the quantities of antimicrobials reported to be used, by antimicrobial use indicators in turkey flocks (*n* = 427 flocks), 2013 to 2019. **(A)** Turkey flock-level milligrams adjusted by population and turkey weight (population correction unit), **(B)** Turkey flock-level number of defined daily doses for animals using Canadian standards adjusted by population, turkey weight at treatment and days at risk, and **(C)** Turkey flock-level number of defined daily doses for animals using Canadian standards adjusted by population and turkey weight (population correction unit).

The flock-level AMU indicators data showed a skewed distribution (Figure 4) as with the broiler chickens. The mean value was higher than the median for mg/PCU_Tk_ but similar for the dose-based indicators. There were also zero values, as with the broiler chickens, for flocks raised as ABF, RWA, organic, or other production types not exposed to antimicrobials (24). Across the studied flocks, the mean antimicrobials reported was 64 mg/PCU_Tk_ (median: 39; minimum:0.15, and maximum: 528). The dose-based indicators also showed between flock variations in nDDDvetCA/1,000 turkey-days at risk and nDDDvetCA/PCU_Tk_ at a mean of 102 (median: 99; 0.19–557) and mean of 9 (median: 8; 0.01–57), respectively.

Descriptive statistics aggregated by AAIs for the weight-based and dose-based AMU indicators are shown in Table 5. Data aggregated by antimicrobial class and the distribution of mg/PCU_Tk_ by antimicrobial class are presented in Supplementary Material 2. The three highest means in mg/PCU_Tk_ were flocks that were medicated with trimethoprim-sulfonamides (*n* = 24 flocks, 109 mg/PCU_Tk_), bacitracins (*n* = 181 flocks; 96 mg/PCU_Tk_), and tetracyclines (*n* = 26 flocks; 62 mg/PCU_Tk_). For the dose based indicators, the relative ranking changed and the three highest means for nDDDvetCA/1,000 turkey-days at risk were noted in flocks that were medicated with trimethoprim and sulfonamides, streptogramins, and bacitracins at 174, 131, and 103, respectively. For nDDDvetCA/PCU_Tk_, the highest means paralleled the previous indicator at 17, 12, and 9 for trimethoprim and sulfonamides, streptogramins, and bacitracins.

#### Identification of High Users of AMU in Turkey Flocks

Flocks defined as high users based on mg/PCU_Tk_ were significantly (*P* ≤ 0.05) more likely to have used antimicrobials in water (*OR* = 3.5) and feed (*OR* = 37.65). These flocks were also significantly (*P* ≤ 0.05) more likely to have used trimethoprim and sulfonamides (*OR* = 7.17), bacitracins (*OR* = 12.31), and tetracyclines (*OR* = 9.96). As well, these flocks were significantly more likely to be in the top 25th percentile of penicillins (*OR* = 5.01) users based on mg/PCU_Tk_. Flocks using streptogramins (*OR* = 0.24) were significantly (*P* ≤ 0.05) less likely to be classified as high users based on mg/PCU_Tk_. It should be noted that all of the antimicrobial classes listed above were administered through multiple routes. It should also be noted that differences were observed between the different marketing weight categories, which may be further explored in future years when data from a larger number of turkey flocks are available.

### *S*election of AMU Indicator and Exploration of Alternative Weight in Denominator for Reporting and Communication

#### Pairwise Correlations Between AMU Indicators

##### Broiler chickens

Pairwise correlation analysis (data from conventional or medicated flocks, *n* = 831) indicated moderate correlations between mg/PCU_Br_ and nDDDvetCA/1,000 broiler-chicken days at risk [Pearson correlation coefficient (PCC) = 0.7039, *P* < 0.001] and between mg/PCU_Br_ and nDDDvetCA/PCU_Br_ (PCC = 0.7503, *P* < 0.001). A significantly high PCC was observed between nDDDvetCA/1,000 broiler-chicken days at risk and nDDDvetCA/PCU_Br_ (PCC = 0.9667, *P* < 0.001).

##### Turkeys

As with broiler chickens, PCC using (data from conventional flocks, *n* = 370) indicated moderate correlations between mg/PCU_Tk_ and nDDDvetCA/1,000 turkey-days at risk (PCC = 0.7062, *P* < 0.001) and between mg/PCU_Tk_ and nDDDvetCA/PCU_Tk_ (PCC = 0.7062, *P* < 0.001). A significantly high correlation was observed between nDDDvetCA/1,000 turkey-days at risk and nDDDvetCA/PCU_Tk_ (PCC = 0.9631, *P* < 0.001).

Tables 6, 7 summarizes the pairwise correlation matrix of the AMU indicators comparing routine CIPARS AMU estimation and using alternate weights in the denominator in broiler chickens and turkeys, respectively. The three pairwise correlation pairs (routine estimations) are also shown in Supplementary Material 2 depicting the highly positive correlation between the two dose-based indicators.

**Table 6:** Pairwise correlation matrix, antimicrobial use indicators in broiler chicken flocks (*n* =831).

**ROUTINE CIPARS AMU ANALYSIS**			
	**Mean**	**Standard error of the mean**	**95% Confidence intervals**
${\text{mg/PCU}}_{\text{Br}}^{\left(\text{CIPARS}\right)}$	150	4	142–159
nDDDvetCA/1,000 broiler chicken-days at risk ^(CIPARS)^	570	13	545–595
${\text{nDDDvetCA/PCU}}_{\text{Br}}^{\left(\text{CIPARS}\right)}$	20	0.5	19–21
**PAIRWISE CORRELATION MATRIX**			
	${\text{mg/PCU}}_{\text{Br}}^{\left(\text{CIPARS}\right)}$	nDDDvetCA/1,000 broiler chicken-days at risk ^(CIPARS)^	${\text{nDDDvetCA/PCU}}_{\text{Br}}^{\left(\text{CIPARS}\right)}$
${\text{mg/PCU}}_{\text{Br}}^{\left(\text{CIPARS}\right)}$	1		
nDDDvetCA/1,000 broiler chicken-days at risk ^(CIPARS)^	0.7039^*^	1	
${\text{nDDDvetCA/PCU}}_{\text{Br}}^{\left(\text{CIPARS}\right)}$	0.7503^*^	0.9667^*^	1
**ALTERNATE AMU ANALYSIS**			
	Mean	Standard error of the mean	95% Confidence intervals
			
${\text{mg/kg}}_{\text{Br}}^{\left(\text{ALT}\right)}$	73	2	70
nDDDvetCA/1,000 broiler chicken-days at risk ^(ALT)^	284	6	271
${\text{nDDDvetCA/kg}}_{\text{Br}}^{\left(\text{ALT}\right)}$	10	0.2	9
**PAIRWISE CORRELATION MATRIX**			
	${\text{mg/kg}}_{\text{Br}}^{\left(\text{ALT}\right)}$	nDDDvetCA/1,000 broiler chicken-days at risk ^(ALT)^	${\text{nDDDvetCA/kg}}_{\text{Br}}^{\left(\text{ALT}\right)}$
${\text{mg/kg}}_{\text{Br}}^{\left(\text{ALT}\right)}$	1		
nDDDvetCA/1,000 broiler chicken-days at risk ^(ALT)^	0.6878^*^	1	
${\text{nDDDvetCA/kg}}_{\text{Br}}^{\left(\text{ALT}\right)}$	0.7000^*^	0.9638^*^	1

*Analysis excluded flocks which were intentionally raised without antibiotics under designated programs for mainstream market such as “Raised without Antibiotics,” “Antibiotic-Free,” and organic*.

*CIPARS—Canadian Integrated Program for Antimicrobial Resistance Surveillance*.

*^(CIPARS)^Based on routine formula used by CIPARS*.

*^(ALT)^kg broiler chicken live pre-slaughter weights, alternate estimation methods*.

*nDDDvetCA—number of defined daily doses for animals using Canadian standards*.

*PCU—population correction unit (based on the European Surveillance for Veterinary Antimicrobial Consumption average weight at treatment for broiler chickens at 1 kg)*.

*Br—broilers*.

* *P < 0.0001, Pearson correlation coefficient*.

**Table 7:** Pairwise correlation matrix, antimicrobial use indicators in turkey flocks (*n* = 370).

**ROUTINE CIPARS AMU ANALYSIS**			
Indicator	Mean	Standard error of the mean	95% Confidence interval
			
${\text{mg/PCU}}_{\text{Tk}}^{\left(\text{CIPARS}\right)}$	75	4	66–83
nDDDvetCA/1,000 turkey-days at risk ^(CIPARS)^	114	4	105–122
${\text{nDDDvetCA/PCU}}_{\text{Tk}}^{\left(\text{CIPARS}\right)}$	10	0.5	9–11
**PAIRWISE CORRELATION MATRIX**			
	mg/PCU_Tk_	nDDDvetCA/1,000 turkey-days at risk ^(CIPARS)^	${\text{nDDDvetCA/PCU}}_{\text{Tk}}^{\left(\text{CIPARS}\right)}$
${\text{mg/PCU}}_{\text{Tk}}^{\left(\text{CIPARS}\right)}$	**1**		
nDDDvetCA/1,000 turkey-days at risk ^(CIPARS)^	0.7062^*^	**1**	
${\text{nDDDvetCA/PCU}}_{\text{Tk}}^{\left(\text{CIPARS}\right)}$	0.7604^*^	0.9631^*^	**1**
**ALTERNATE AMU ANALYSIS**			
	Mean	Standard error of the mean	95% Confidence interval
${\text{mg/kg}}_{\text{Tk}}^{\left(\text{ALT}\right)}$	50	2	45–54
nDDDvetCA/1,000 turkey-days at risk ^(ALT)^	86	3	79–92
${\text{nDDDvetCA/kg}}_{\text{Tk}}^{\left(\text{ALT}\right)}$	7	0	7–8
**PAIRWISE CORRELATION MATRIX**			
	${\text{mg/kg}}_{\text{Tk}}^{\left(\text{ALT}\right)}$	nDDDvetCA/1,000 turkey-days at risk ^(ALT)^	${\text{nDDDvetCA/kg}}_{\text{Tk}}^{\left(\text{ALT}\right)}$
${\text{mg/kg}}_{\text{Tk}}^{\left(\text{ALT}\right)}$	**1**		
nDDDvetCA/1,000 turkey-days at risk ^(ALT)^	0.5376^*^	**1**	
${\text{nDDDvetCA/kg}}_{\text{Tk}}^{\left(\text{ALT}\right)}$	0.5727^*^	0.8662^*^	**1**

*Analysis excluded flocks which were intentionally raised without antibiotics under designated programs for mainstream market such as “Raised without Antibiotics,” “Antibiotic-Free,” and organic*.

*CIPARS—Canadian Integrated Program for Antimicrobial Resistance Surveillance*.

*^(CIPARS)^Based on routine CIPARS formula*.

*^(ALT)^kg turkey live pre-slaughter weights, alternate estimation methods*.

*nDDDvetCA—number of defined daily doses for animals using Canadian standards*.

*PCU—population correction unit (based on the European Surveillance for Veterinary Antimicrobial Consumption average weight at treatment for turkeys at 6.5 kg)*.

*Tk—turkeys*.

* *P < 0.0001, Pearson correlation coefficient*.

#### Exploration of Alternate Weights in the Denominators

##### Broiler chickens

When the input parameter in the denominator was changed to the actual kg broiler chicken biomass recorded at the time of the pre-harvest visit or the pre-slaughter live weight (mean of 2 kg; 1.2–4.4) instead of the ESVAC's average weight at treatment of 1 kg, the estimates of use decreased by ~50% (Table 6).

It is important to note (Table 6) that the change in denominator to kg live broiler chicken biomass resulted in a slight decrease in PCC, though it remained moderate between the weight-based mg/kg vs. the two dose-based indicators nDDDvetCA/1,000 broiler chicken-days at risk (PCC = 0.6951, *P* < 0.0001) and nDDDvetCA/PCU (PCC = 0.7058, *P* < 0.0001). Correlation between the two dose-based indicators remained significantly high (PCC = 0.9648, *P* < 0.0001).

##### Turkeys

Using actual kg live turkey biomass (mean weights: all categories [10 kg], broiler turkeys [5 kg], light hens [7 kg], heavy hens [9 kg], light toms [12 kg], heavy tom [15 kg]) at the time of the pre-harvest visit instead of the ESVAC's average weight at treatment of 6.5 kg (Table 3), decreased the estimates by ~33% unlike the broiler chickens data where reduction was by 50% (Table 7).

Unlike the broiler chickens, the change in denominator to kg live turkey biomass had a greater impact on the PCC values (Table 7). Pearson correlation coefficients decreased between the weight-based mg/kg vs. the two dose-based indicators nDDDvetCA/1,000 turkey-days at risk (PCC = 0.5376, *P* < 0.0001) and nDDDvetCA/PCU (PCC = 0.5727, *P* < 0.0001). Correlation between the two dose-based indicators slightly decreased but remained significantly high (PCC = 0.8662, *P* < 0.0001; Table 7).

### Overall AMU by Route of Administration and National Temporal Trends

For broiler chickens, when the quantitative data from all years were combined, the highest proportion of antimicrobials reported were those administered via the feed (92%) and smaller proportion was administered via water (8%) and injections (<1%). Similar proportions of antimicrobials reported were noticed for turkeys for feed (96%), water (8%), and injections (1%). Over time, the proportion of use by route of administration remained consistent until 2018 (Supplementary Material 2) where the proportion of antimicrobials administered via water increased from 5 to 14% in broiler chickens and from 3 to 11% in turkeys. Antimicrobials administered via injection constituted <1% of the total AMU and frequency of this use decreased over time; a small quantity of injectable antimicrobial, lincomycin-spectinomycin, was administered in broiler chicks and gentamicin in turkey poults at the hatchery via injections in 2019. One turkey flock in 2013 was treated with ceftiofur *in ovo* at the hatchery.

**Figure 5:**
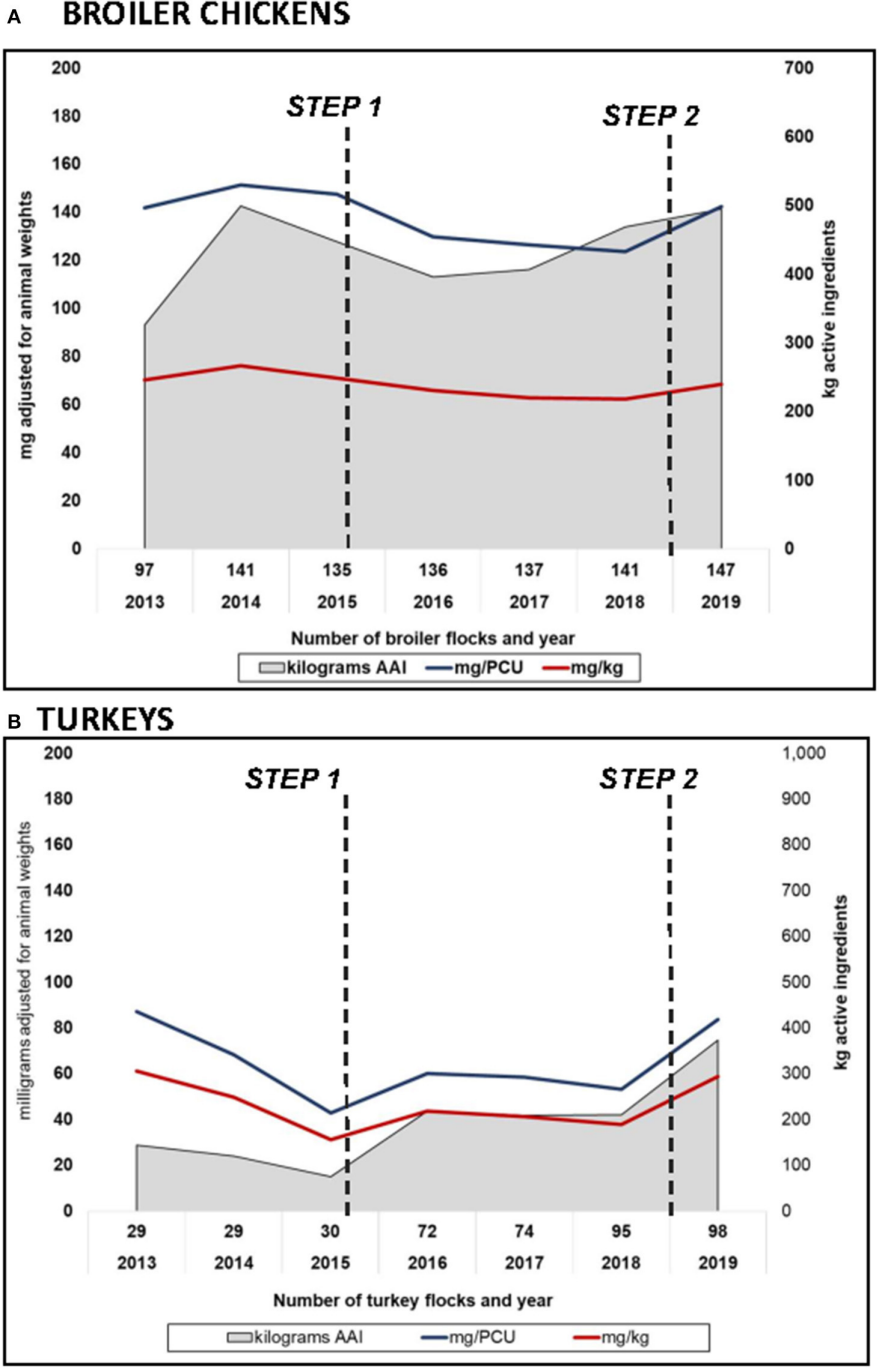
Temporal trends in reported antimicrobial use in **(A)** broiler chickens and **(B)** turkey flocks using routine CIPARS estimation methodology and alternative biomass calculations, milligrams per population correction unit (mg/PCU) using ESVAC's average weight at treatment (mg/PCU), and alternate biomass estimation using milligrams per kg live pre-slaughter weight or animal biomass. *2013 to 2015 data in turkeys pertain to British Columbia (initial surveillance site). Steps 1 and 2 correspond to the industry antimicrobial use reduction strategy*.

**Figure 6:**
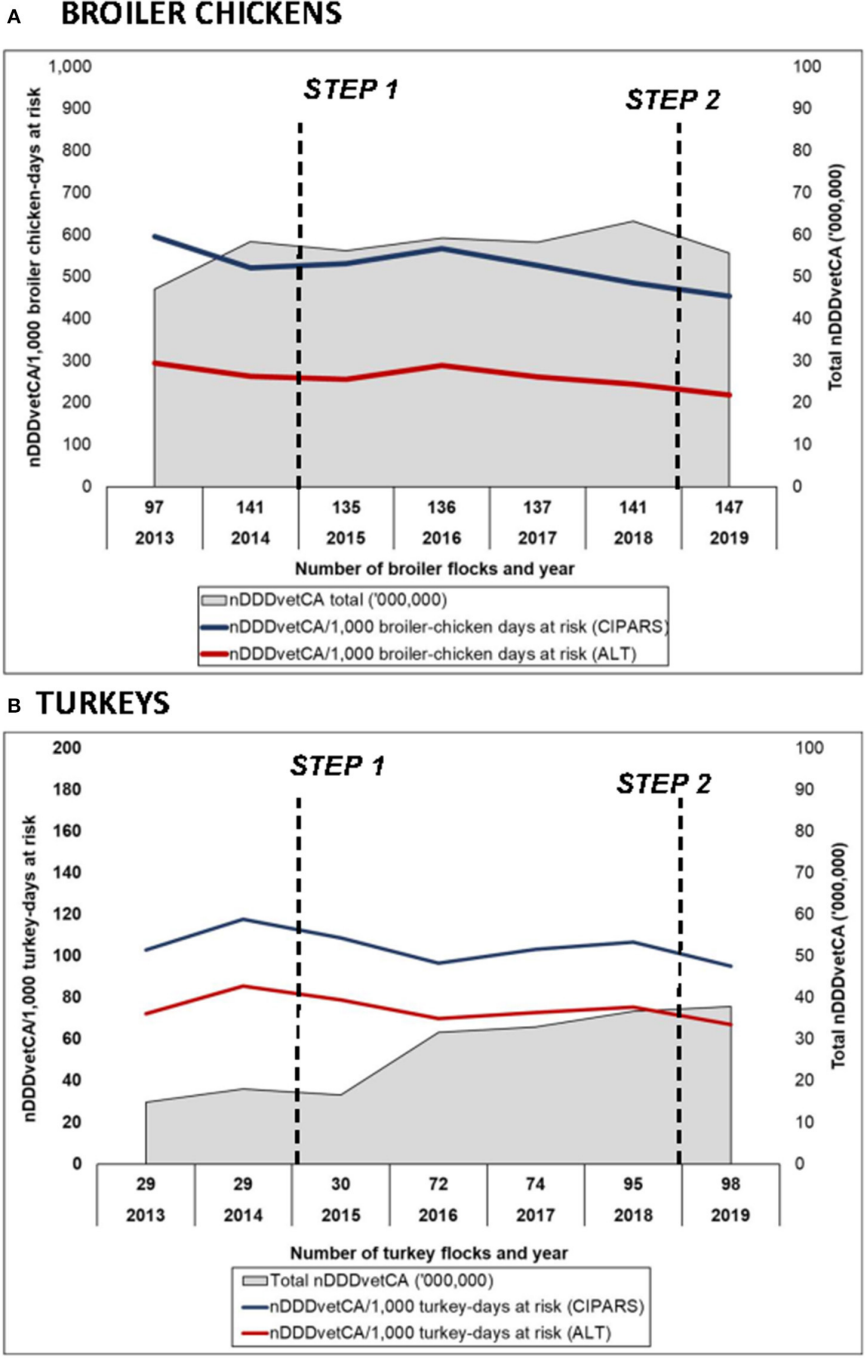
Temporal trends in reported antimicrobial use in **(A)** broiler chickens and **(B)** turkey flocks using routine CIPARS estimation methodology and alternate estimation, number of defined daily doses in animals using Canadian standards per 1,000 animal-days at risk. *2013–2015 data in turkeys were British Columbia (initial surveillance site). Steps 1 and 2 correspond to the industry antimicrobial use reduction strategy*.

For trends over time, estimates using routine CIPARS methodology and using alternate weights in the denominator showed relatively similar trends but as anticipated, a lower magnitude using the pre-harvest weights for both broilers and turkeys (Figure 5). Similar trends were observed in the dose-based indicators (nDDDvetCA/1,000 broiler-chicken or turkey-days at risk). It is important to note that this latter indicator, which corrects for dose showed a decrease in 2019 unlike the weight based indicator (Figure 6) due to the shift in the AAIs that constituted overall use for that year in both species (i.e., shift from AAIs with low DDDvetCA's to AAIs with relatively higher DDDvetCA's).

## Discussion

Building on our methodology for estimating farm-level national AMU data (7, 19) this study further explored AMU characteristics and the utility of different AMU indicators at the flock-level with the intent of informing best practices for surveillance analysis and reporting. We demonstrated how the application of quantitative AMU indicator (mg/PCU) and qualitative AMU metrics (frequency of use, route of administration) complement each other in characterizing high users of antimicrobials. We envisaged that this approach will enhance the methodology for producer and veterinarian reporting and for providing feedback to the poultry industry. Our data indicated that high users were those that used antimicrobials via water and certain classes indicated for the therapy of systemic diseases in poultry; these are in addition to a routine necrotic enteritis program.

We determined that the three AMU indicators currently used by CIPARS were moderately or closely related, suggestive of the necessity of using at least two indicators, one weight-based and one dose-based, to better characterize the evolving AMU patterns associated with current AMU reduction initiatives in broilers and turkeys in Canada (16, 17). Finally, we have shown that a change to the input parameter in the denominator did not impact reported AMU distribution and AMU trends. Thus, alternate choices for weight of the birds could be considered for their utility for national surveillance reporting, evaluated for relevance in the Canadian industry context and well as their uptake by veterinarians and producers.

This study provided new and detailed information about AMU in Canadian broiler chickens and turkeys by exploring data at the flock level. We previously described the use of the weight-based (mg/PCU) and dose-based (nDDDvetCA/1,000 animal-days at risk and nDDDvet/PCU) indicators in the aggregated farm AMU data and concluded that the interpretation of the results could change depending on the indicator chosen (19, 23). In particular, the relative ranking of the antimicrobial classes changed, depending on the indicator chosen, similar to another poultry AMU study in France (25). Our data also showed variations in temporal trends between the dose-based and weight-based indicators. However, in terms of flock distribution, the 3 AMU indicators showed similar patterns (i.e., all skewed distribution), indicative of the overall range of production practices and evolving AMU patterns of use in Canadian poultry.

The flocks raised as RWA, ABF, and organic flocks (i.e., no exposures to any antimicrobials) were excluded from the analysis evaluating the correlation between the AMU indicators. The decision not to use antimicrobials in these flocks was not based on flock-level parameters such as mortality, health status, or chick source, but instead on program-level requirements (i.e., market-driven). Differences in flock-level parameters between conventional flocks and flocks participating in these programs have been explored in other research (26).

CIPARS currently does not conduct benchmarking of AMU, but provides feedback on AMU to the industry and to participating producers and veterinarians, in addition to generating national AMR and AMU estimates. It is acknowledged that dose-based AMU indicators are utilized in other countries for benchmarking purposes (22, 27, 28). For comparability with other animal species (i.e., where DDDvetCA standards are yet to be developed) and other sources of AMU data (e.g., national sales and distribution data for terrestrial and aquatic animals) within Canada, we utilized the weight-based indicator, mg/PCU to identify high users, and complemented this with other qualitative data collected through our questionnaire. In our present study, there were diverse AMU patterns utilized by broiler chicken and turkey producers. Our analysis indicated that high users were significantly more likely to have used antimicrobials in water and specific classes including bacitracins, penicillins, tetracyclines, and trimethoprim-sulfonamides. Analysis in turkeys yielded similar results with the exception of aminoglycosides. As we have previously described (29), conventional flocks were typically fed with AAIs efficacious against Gram positive organisms, primarily *Clostridium perfringens*, the causative agent of necrotic enteritis. It can be inferred that the high users administered other antimicrobials in addition to their preventive necrotic enteritis program in the face of a clinical condition or bacterial disease outbreak (and may be explained by the flock AMU profiles with ≥3 AAIs). The classes associated with high use were those that have broad-spectrum of activity and are indicated for the treatment of systemic bacterial infections (30). Bacitracin was also associated with high use and could be due to the following reasons: (1) higher inclusion rate in feed for “reduction of early mortality due to diminished feed consumption and chilling” (11), and (2) evolving patterns of use in the poultry industry as a result of the AMU reduction strategy (increased use of VDD's medium important antimicrobial classes such as bacitracins). The prophylactic use of antimicrobials and other farm-level factors (e.g., AMU decisions by the producer or farm staff) have been identified as a risk factor for high use in turkeys (31).

It is important to note that the other classes used for the prevention of necrotic enteritis such as, macrolides, streptogramins, and orthosomycins were found to be associated with low users of antimicrobials. The approved level of drug or inclusion rates in g/ton of feed for these classes are relatively lower (11). Except avilamycin, the preventive use of these antimicrobial classes was eliminated at the end of 2018. Hence, AMU patterns may continue to evolve and the reported quantity of use could further change over time.

Taken together, the complementarity of a quantitative AMU indicator and qualitative AMU metrics in identifying high users are essential variables in understanding the dynamics of AMU practices. For providing feedback to veterinarians and producers, it may be useful to identify and highlight those flocks that used treatment via water and classes other than those that are indicated for necrotic enteritis. Our future analysis will investigate the factors associated with high and medium-to-low users of antimicrobials based on DDDvetCA/1,000 chicken-days at risk. This will address the effect of the type of antimicrobial reported to be used on the results. As in another poultry study (31), additional flock health (e.g., vaccinations and non-antimicrobial alternatives), biosecurity, and farm-level demographic factors will also be included in these future analyses in order to better understand the drivers of higher use in broiler chicken and turkey flocks in Canada. Using input parameters already collected by CIPARS (e.g., treatment frequency, duration of treatment/days of exposure, weight at actual treatment), future work could explore other potential dose-based AMU indicators to use for identifying high users of antimicrobials.

For the purposes of surveillance reporting at both the national and veterinarian-producer level, we explored how the AMU indicators are correlated in order to facilitate the selection of indicator(s). By investigating the degree of correlation between the multiple metrics, we have shown that using different numerators is quite informative. Due to the use of the same input parameters (i.e., formulaically the same), data reported using the weight-based and dose-based indicators will be necessarily correlated. One source of variation between results reported by different AMU indicators is the AAI, which could vary by dose, duration of exposure, weight at treatment and reasons for use. The dose-based indicator, nDDDvetCA/1,000 animal days at risk accounts for both population and days at risk of being exposed to antimicrobials. The days at risk depends on production type and life span of the bird, for example, it is shorter in broiler chickens [our study and a similar broiler AMU study (21)] and longer in small holder chicken flocks (8) or turkeys (our study). The CIPARS farm-level AMU data is based on one grow-out cycle from sentinel farms unlike some other farm-level AMU monitoring programs where continuous full-year (i.e., the data collection period at risk) data are collected (5). This could limit the ability to compare our data with that from other surveillance programs or poultry studies that do not have a similar design or the same period at risk. However, our analysis indicated that the two dose-based indicators, nDDDvetCA/1,000 animal-days at risk and nDDDvetCA/PCU showed high correlation indicating that either of these could be used for characterizing temporal trends and facilitate comparability with other surveillance programs. This is particularly important, since sampling from one grow-out period vs. the entire year may require fewer resources and be more attainable for some countries. The choice of which indicator to use should consider stakeholder understanding, relevance to stakeholders, or preference and availability of the input parameters required, such as days at risk. The nDDDvetCA/PCU (4) could be used in datasets or data collection points where the time component is unavailable or constant (e.g., annual aggregated sales data), or in smaller scale targeted studies (32).

For characterizing national temporal trends, we explored the use of different weights in the denominator, as the reported antimicrobial use estimates for a specific indicator could also change depending on the input parameters (9). In the present study, the mg/PCU and analysis using alternate biomass using the broiler chicken and turkey live pre-slaughter weight, showed similar temporal trends, indicative that the choice of which weight in the denominator to use is a preference, which affects the magnitude of the measure, but will not alter the reported trends; provided that a consistent weight is applied over time.

The 2 kg average weight for broiler chickens at slaughter was within the industry standards for the commonly raised breeds in Canada at 34–35 days. This weight is double the ESVAC average weight at treatment of 1 kg. Using the 2 kg weight in the denominator consequently reduced the magnitude of the AMU estimates by 50%. Whereas for turkeys, because of the different marketing weight categories and higher proportion of heavy bird categories, the average kg at slaughter was closer to the ESVAC average weight at treatment of 6.5 kg, yielding smaller differences between mg/PCU and mg/kg. In a similar study in pigs, changing an input parameter in the denominator (9) did not impact the distribution of AMU, as the choice of weight is simply a different scaler variable in the denominator applied equally to the numerator for each antimicrobial.

For communication with producers, veterinarians and the industry in general, the mg/kg live pre-slaughter weight might be preferable because it might be easier to understand. For example, “results pertain to mg of AAIs used for every kg of chicken or turkey live-weight shipped for slaughter during growing cycle A” or nDDDvet/kg could be expressed as “results pertain to the total number of doses used for every kg of chicken or turkey live weight shipped for slaughter during the growing cycle B.” However, the kg pre-slaughter weight, driven by specific market weight preferences, potential disease conditions, and change in genetics or nutrition requirements could also vary over time. The stability of this measurement needs to be considered. The mean weights at treatment were also characterized in this study; it is important to note that the mean weights at treatment in our dataset were 0.84 and 3.0 kg for broiler chickens and turkeys, respectively. The mean treatment weights varied over time and also related to the evolving AMU practices in the industry, specifically, the removal of the preventive use of certain AAIs belonging to higher VDD categories and typically used in younger birds (i.e., injection of ceftiofur, lincomycin-spectinomycin, and gentamicin at day of hatch). With the full implementation of the AMU reduction strategy, it is conceivable that AMU practices could further change. In particular the practice of continuous administration of AAI via feed for prevention beginning at day 1 (chick or poult placement) is expected to shift toward targeted treatment when birds are most likely to be susceptible to enteric and respiratory diseases or only when deemed necessary. From an AMU stewardship standpoint, in our circumstance, the average actual weight at treatment may not also be a stable denominator to use for characterizing temporal trends as it potentially influence the accuracy of reflecting true use changes over time, which are critical for monitoring the impact of an AMU intervention.

Overall, our analysis indicated that the quantity of antimicrobials used in broiler chickens and turkeys in Canada was relatively higher compared to poultry in Europe, for example, Sweden (33) and the United Kingdom (34) in terms of mg/kg, and Belgium (21) in terms of nDDDvetCA/1,000 animal-days at risk (or treatment incidence). Water treatment and the use of certain classes (trimethoprim-sulfonamides, tetracyclines) were associated with high use of antimicrobials, thus underlying factors (e.g., coinfection with emerging viral diseases, barn-level factors) contributing to the diseases targeted by the classes implicated with high use warrants further research. The decreasing diversity of AAIs in more recent years (2018–2019) is indicative of evolving AMU patterns of use related to voluntary decisions by the poultry industry. The industry AMU strategy called for the elimination of the preventive use of at least 5 antimicrobial classes. However, the effect of the shift from prevention to treatment uses needs to be monitored; as these would still contribute to the overall quantity of AMU. At the national level, the interpretation of overall AMU could change depending on the indicator chosen, particularly, the change in the relative ranking of the classes and temporal trends. The dose-based indicator corrects for differences in AMU classes and/or practices, thus enabling between-farm comparisons and better detection of temporal shifts in AMU. This further emphasizes the need for more than one AMU indicator in characterizing the flock-level and national level AMU dynamics in the poultry industry. Finally, we have shown that a change in the denominator (animal biomass) will impact the magnitude of the measure but will not alter the trends provided that a consistent weight is applied. The choice of the weight should reflect the surveillance system objectives; which could be to facilitate reporting back to farmers/veterinarians (i.e., reflective of their preference for understanding and uptake) or for international reporting (creating an appropriate comparison), or both. Stakeholder consultations to explore reporting preferences and the development of an algorithm for identifying high users for farm-level reporting are necessary next steps.

## Data Availability Statement

All datasets presented in this study are included in the article/Supplementary Material.

## Ethics Statement

No animal studies/experiments included in the manuscript. Data were extracted from the CIPARS database. No personal identifiers linking the producers, farm location, or veterinarian were collected. Data pertains to the animals and obtained from farm records and actual farm visit. An informed consent form was administered by the veterinarian to the producer prior to the farm visit.

## Author Contributions

AA, AD, SG, DL, and CC conceived the study. AA and AD conducted the formal analysis. AB, DL, and CC developed the DDDvetCA methodology/assignment. SG, SK, and AA contributed to the ongoing refinements to the AMU database, data curation, and data validation. RR-S, AA, SG, DL, and AD acquired partial funding. RR-S provided overall technical supervision and farm program operation. CC, DL, SG, AD, AB, SK, and AA contributed to the writing, editing, and review of the manuscript. All authors contributed to the article and approved the submitted version.

## Conflict of Interest

The authors declare that the research was conducted in the absence of any commercial or financial relationships that could be construed as a potential conflict of interest.

## Supplementary Material

Supplementary Material 1 Antimicrobial use patterns in broiler chickens and turkeys during the study timeframe (2013–2019).

Supplementary Material 2 Example, farm-level antimicrobial use questionnaire (2019 version).

Supplementary Material 3 Other findings. Other antimicrobial use descriptive statistics and correlation matrices.
