# Supplementary material for: Antimicrobial Use Indices—The Value of Reporting Antimicrobial Use in Multiple Ways Using Data From Canadian Broiler Chicken and Turkey Farms
Source: Front Vet Sci. 2020 Oct 19;7:567872. doi: 10.3389/fvets.2020.567872 (PMC7604299; doi:10.3389/fvets.2020.567872)
Supplement: Supplementary Material 2 — Example, farm-level antimicrobial use questionnaire (2019 version). [file Data_Sheet_2.pdf]

BROILER FARM QUESTIONNAIRE	Hatchery ID Code:	
FULL BROILER GROW-OUT PERIOD	Flock ID Code:	BCSF
	VET ID Code:	BCSV

CIPARS/FOODNET CANADA.FC.2019

CIPARS AND FOODNET CANADA[1] FARM SURVEILLANCE
BROILER CHICKENS

INSTRUCTIONS PLEASE READ CAREFULLY:

- ❑ This questionnaire is to be completed **during the last week of grow before slaughter, (> 30 days of age).**
- ❑ Enter the **Hatchery ID, Flock ID and VET ID** code in the box on the top right corner of **each page**.
- ❑ **Hatchery ID** will be provided by the Canadian Poultry and Egg Processors Council (CPEPC) to all participating veterinarians.
- ❑ **Please answer all questions; please call the hatchery to confirm hatchery-related information.**
  If the information is unknown, indicate "**D/K**" (Don't know) as the response.
  If a question is not applicable, indicate "**N/A**" as the response.
- ❑ Note that certain information required is at **BARN LEVEL** and **FLOOR LEVEL**. These are **EXTREMELY** critical for our analysis.
- ❑ This questionnaire is in triplicate.
  White copy: Send to CIPARS in the FEDEX envelope provided;
  Yellow copy: Retained by the field worker/poultry veterinarian;
  Pink copy: Retained by the producer.

If you are unclear on how to answer any question please feel free to contact the CIPARS Farm Working Group

THANK YOU FOR YOUR PARTICIPATION!

[1] CIPARS – Canadian Integrated Program for Antimicrobial Resistance Surveillance, a national program that monitors antimicrobial use and resistance.
FoodNet Canada (formerly known as C-EnterNet) is a multi-partner sentinel site surveillance program that includes simultaneous and in-depth investigation of food-borne and waterborne diseases and exposure.

**BROILER FARM QUESTIONNAIRE**	**Hatchery ID Code:**
**FULL BROILER GROW-OUT PERIOD**	**Flock ID Code:** **BCSF**
	**VET ID Code:** **BCSV**

CIPARS/FOODNET CANADA.FC.2019

Please complete the table below for the pre-harvest samples collected today:

Please Note: Collect at least 15 fresh caecal droppings per pooled sample; each sample should represent one quadrant of the floor space.

	SAMPLE ID CODE
Sample #1	
Sample #2	
Sample #3	
Sample #4	

BROILER FARM QUESTIONNAIRE	Flock ID Code: BCSF
FULL GROW-OUT PERIOD	VET ID Code: BCSV

CIPARS/FOODNET CANADA.FC.2019

NOTE: ***All data including inventory and feed tonnage for this questionnaire should be provided for the BARN AND FLOOR LEVELS.***

1. **Province in which this flock is located:**

   ❍ British Columbia ❍ Alberta ❍ Saskatchewan ❍ Manitoba ❍ Ontario ❍ Québec ❍ Atlantic

2. **Date of chick placement:** ____ / ____ / ____ (Month / Day / Year)

3. **Pre-harvest sample collection date:** ____ / ____ / ____ (Month / Day / Year)

4. **Age at day of sampling:** ____________ Days

5. **Average weight at day of sampling:** ____________ Kilograms

6. **Quota period:** A-____________

7. **Floor space (barn):** ____________ Square ft.

8. **Stocking density** (floor space/total **placed**) ____________ Sq. ft./bird

9. **Total farm capacity** ____________ birds

If there are multiple barns on this site then please indicate the barn name or number (for your reference)**:** ____________

If this is a multi-floor barn, please indicate which floor was sampled today (for your reference)**:**

❍ Top floor ❍ Middle floor ❍ Bottom floor

<u>Farm level tracking</u>**: If this barn or floor was sampled previously, please indicate**:

Year sampled: ______________________ **Flock code:** ________________________________

10. Flock Information

A. Do you consider your broiler operation to be: (check all that apply)

❑ All-In-All-Out ❑ Multi-age ❑ Multi-species or multi-commodity

B. Broiler strain: (check all that apply for the BARN)

❑ Ross x Ross *(check all applicable)*
- ❑ 308
- ❑ 708
- ❑ Others/unknown: __________________

❑ Cobb x Cobb *(check all applicable)*
- ❑ 500
- ❑ 700
- ❑ Others/unknown: _________________

❑ Other strains: specify (e.g., Hubbard) _______________

C. Are these birds marketed as: (check only one):

❍ Conventional *(exposed to antimicrobials, ionophores or chemical coccidiostats)*
❍ Antibiotic-free **(ABF)** or Raised Without Antibiotics (RWA)
- ❍ NO antibiotics, ionophores, chemical coccidiostats (**full/mainstream program**)
- ❍ Treated with Ionophores only
- ❍ Treated with Chemical coccidiostats only
- ❍ Other (please specify): _________________

❍ Organic
❍ Other: ________

D. If growing **ABF regularly,** indicate months and/or years in the program

____
(Months) (Years)

Certifying body, if any: ____________________________________

D.1 If flocks are **not** routinely managed as antibiotic free , please check here ❍

11. Flock inventory for this BARN AND FLOOR (these data are critical for our analysis)

	BARN TOTAL	FLOOR*	
**A.** Initial population (total **CHICKS** placed)			Chicks
**B.** Final flock population (total **GROWN BIRDS** on day of sampling)			Birds
**C.** Percent mortality **(A – B) ÷ A x 100**:			%

***Population in floor to be sampled today (if multi-floor barn)**

12. HATCHERY: Breeder Flock Source Information

Please call the hatcheries to confirm breeder flock source - **NOTE**: These data are critical for our analysis

**Flock source origin**	**Percent of chicks placed**	**Age range of breeder flock source***		**Purchased by hatchery as** (Check all that apply)**:**	
		Youngest	Oldest	Chicks	Hatching Eggs
**DOMESTIC** *(circle province)*:<br>BC AB SK MB ON QC Atlantic	%	weeks	weeks	❑	❑
**DOMESTIC – OTHER PROVINCES** *(circle province)*:<br>BC AB SK MB ON QC Atlantic	%	weeks	weeks	❑	❑
**IMPORTED:**	%	weeks	weeks	❑	❑
	**100 %**				

*Please use this as a guide if hatchery uses the following description: **young flocks** (or small chicks) - 30 wks and younger; **prime flocks** (or medium/standard chicks) – 31 to 50; **old flocks** (or large chicks) – 51 and older.

13. Hatchery Drug Use – Please indicate drug(s) used at the hatchery for this growing cycle. **Please call the hatchery to confirm drug use, dose and reasons for use. These information are critical for our analysis.**

Check here ❍ if NO antimicrobials were given at the hatchery.

Antimicrobial	Brand name	**Route** (choose only one)	Dose per egg or chick	Proportion of chicks medicated
Ceftiofur	Excenel®	❍ *In ovo*<br>❍ Subcutaneous<br>❍ *Per os*	(circle) µg, mg, ml<br>**Routine: 0.10 to 0.20 mg/egg or chick**	❍ 1-25% ❍ 26-50%<br>❍ 51-75% ❍ 76-100%
Enrofloxacin	Baytril®	❍ *In ovo*<br>❍ Subcutaneous<br>❍ *Per os*	(circle) µg, mg, ml	❍ 1-25% ❍ 26-50%<br>❍ 51-75% ❍ 76-100%
Gentamicin	Gentocin®	❍ *In ovo*<br>❍ Subcutaneous<br>❍ *Per os*	(circle) µg, mg, ml<br>**Approved dose: 0.20 mg/egg or chick**	❍ 1-25% ❍ 26-50%<br>❍ 51-75% ❍ 76-100%
Lincomycin-Spect.	Linco-Spectin®	❍ *In ovo*<br>❍ Subcutaneous<br>❍ *Per os*	(circle) µg, mg, ml<br>**Routine: 0.75 mg/egg or chick***	❍ 1-25% ❍ 26-50%<br>❍ 51-75% ❍ 76-100%
Others (*please specify*)		❍ *In ovo*<br>❍ Subcutaneous<br>❍ *Per os*	(circle) µg, mg, ml	❍ 1-25% ❍ 26-50%<br>❍ 51-75% ❍ 76-100%

* based on 11 mg/kg body weight (or egg/chick weight equivalent) or **0.75 mg/chick consisting of 0.5 mg spectinomycin and 0.25 mg lincomycin.**

14. Reasons for Drug use- Please indicate reasons for drug use

**Antimicrobial** (from #13, e.g., ceftiofur)	**Primary Reason for Medication Use** (Please choose **only one primary reason**)	**Please indicate the target disease for this antimicrobial use** (Please check all that apply)
	❍ Disease Prevention<br>❍ Disease Treatment<br>❍ Producer Request<br>❍ High risk breeder flock source (e.g., young, old)	❑ *E. coli*<br>❑ *Enterococcus cecorum*<br>❑ *Salmonella*<br>❑ *Staphylococcus*<br>❑ Early clostridial infections<br>❑ Other diseases (*please specify*):

15. **Hatchery-level vaccinations** (day18 of embryonation or at hatch ONLY for this section). Please check all of the vaccines administered at the hatchery.

Check here ❍ if NO vaccines/bacterins administered at the hatchery.

Agent	Trade names/brand, manufacturer	
Coccidiosis vaccines	❑ Coccivac-B52 (Merck AH)<br>❑ Coccivac-D2 (Merck AH)<br>❑ Hatchpak Cocci III (Merial-BI)	❑ Immucox for chickens I (Ceva AH)<br>❑ Immucox for chickens II (Ceva AH)<br>❑ Others, *please specify:* ______________
Bronchitis vaccines	❑ Bronchitis Vaccine, Mass. (Zoetis)<br>❑ Bronchitis Vaccine, Mass. freeze-dried (Merial-BI)<br>❑ IB Vac H, Mass. Holland Strain (Merck AH)	❑ Mildvac Ma5, Mass. (Merck AH)<br>❑ Bronchitis Vaccine, Mass. + Conn. (Zoetis)<br>❑ Others, *please specify:* ______________
Infectious Bursal Disease	❑ 89/03, variant, frozen (Merck AH)<br>❑ Avipro Vibursa CE (LAHI)<br>❑ Bursal Disease S-706 (Merial-BI)<br>❑ Bursa Blen M (Merial-BI)<br>❑ Bursavac (Merck AH)<br>❑ Bursimune (Ceva AH)<br>❑ Bursine 2 (Zoetis)	❑ Clonevac D78 (Merck AH)<br>❑ Poulvac Bursaplex (Zoetis)<br>❑ SVS 510 (Merial-BI)<br>❑ Univax BD (Merck AH)<br>❑ Univax Plus (Merck AH)<br>❑ Others, *please specify*:______________
Marek's Disease	❑ Cevac MD-HVT (Ceva AH)<br>❑ Marek's HVT (Merial-BI)<br>❑ MD-VAC Frozen (Zoetis)<br>❑ Others *please specify:* ________________	
Marek's Disease + Infectious Bursal Disease	❑ Bursal Disease-Marek's Disease (Merial-BI)<br>❑ Vaxxitek HVT+IBD (Merial-BI)<br>❑ Vectormune HVT+IBD (Ceva AH)<br>❑ Others *please specify:* _________________	
Marek's Disease + Newcastle Disease	❑ Innovax, MD Vectored (Merck AH)<br>❑ Others *please specify:* __________________	
Reovirus	❑ Enterovax (Merck AH)<br>❑ Others *please specify, e.g., autogenous:*__________________	
Bacterial organisms	❑ *Salmonella*, Avipro MeganVAC1 (LAHI)<br>❑ *Salmonella,* Poulvac ST (Zoetis)<br>❑ *Salmonella*, Salmune (Ceva AH)<br>❑ *E. coli*, Poulvac E. coli (Zoetis)<br>❑ Others *please specify* :__________________	

16. Level of Biosecurity.

1. Access Management		
Presence of livestock and poultry within a 1 km radius or within farm premises	❍No	❍Yes – please specify<br>❑ Cattle ❑ Broilers<br>❑ Pigs ❑ Broiler breeders<br>❑ Turkeys ❑ Hatchery<br>❑ Layers<br>❑ Others: ______________________
Presence of domestic (non-livestock) and wild animals on-farm (as observed at the time of visit)	❍No	❍Yes
		❑ Dogs ❑ Wild birds<br>❑ Cats ❑ Wild animals (raccoons, foxes, others)<br>❑ Horses<br>❑ Others: ___________________________
Recognizable biosecurity zones (controlled access zone delineated from restricted access zone)	❍No	❍Yes
Foot bath/boot dip	❍No	❍Yes
Danish entry system (has a detailed barn entry protocol)	❍No	❍Yes
Personal protective equipment required for access to production area	❍No	❍Yes (check all that apply)
		❑ Boots ❑ Gloves ❑ Coveralls/Designated farm clothes
**2. Animal health management**		
Downtime[2] between flocks	❍No	❍Yes: Days:
**3. Operational management**		
Daily dead bird collection/removal from production area	❍No	❍Yes
Manure stored within farm premise	❍No	❍Yes<br>❑ Adjacent to barns<br>❑ Designated storage within the production area (controlled access zone). **If stored on-farm, please specify:**<br>**STORAGE DURATION**:____________________(days)<br>❑ Others__________
Manure removal (check all applicable)		❑ Removed from barn under nutrient management plan<br>❑ On-farm composting<br>❑ Spread on field adjacent to production area (within 0.5 km)<br>❑ Spread on field >1 km away by producer<br>❑ Spread on field elsewhere by contracted services<br>❑ Others:___________

If spread on field by producer, circle approximate spreading months:

JAN – FEB – MAR – APR – MAY – JUN – JUL – AUG – SEP – OCT – NOV - DEC

Level of Biosecurity, continued

Integrated pest control program	❍No	❍Yes - please specify **pest program**:
		❑ **Rodents**<br>*check all applicable*: ❑ bait stations ❑ traps ❑ others ___________________<br>❑ **Beetles**<br>*check all applicable*: ❑ insecticide ❑ others _____________________________<br>❑ **Wild birds**<br>*check all applicable*: ❑ traps ❑ screenings ❑others______________________<br>❑ **Flies**<br>*check all applicable*: ❑ fly screens ❑ traps ❑ insecticides<br>❑others____<br>❑ **Other programs**:____
Premise sanitation<br>(before this cycle)	❍No	❍Yes – check all applicable
		❑ Washed<br>❑ Hot water washed<br>❑ Dry clean<br>❑ Dry clean & washed<br>❑ Dry clean and hot water washed<br>❑ Other: ________
Disinfection<br>(NOTE: information required is before this cycle only, not for the year)	❍No	❍Yes – please specify **chemical used**: *(you may use **Appendix 1** for reference)*
		❑ **Quat**, *by:* ❑ flood (↓ pressure) ❑↑pressure ❑ fog/thermofog<br>❑ **Aldehydes,** *by:* ❑ flood (↓ pressure) ❑↑pressure ❑ fog/thermofog<br>❑ **Phenol,** *by:* ❑ flood (↓ pressure) ❑↑pressure ❑ fog/thermofog<br>❑ **Chlorine-based**, *by:* ❑ flood (↓ pressure) ❑↑pressure ❑ fog/thermofog<br>❑ **Iodine**, *by*: ❑ flood (↓ pressure) ❑↑pressure ❑ fog/thermofog<br>❑ **Multi-ingredient product**, ❑ flood (↓ pressure) ❑↑pressure ❑ fog/thermofog<br>❑ **Other:** ___________, *by:* ❑ flood (↓ pressure) ❑↑pressure ❑ fog/thermofog<br>❍ **Don't know**
Water source		❑ Municipal<br>❑ Well water<br>❑ Dug (excavated below the ground water table, ~ 10 to 30 feet deep)<br>❑ Drilled shallow (≤ 100 feet deep)<br>❑ Drilled deep (≥ 100 feet deep)<br>❑ Other: _____<br>❑ Other (lakes, rivers, ponds, cisterns):________________
Water analysis done regularly	❍No	❍ Yes – please specify **frequency of testing:**
		❍ Monthly ❍ 2x yearly ❍ Yearly ❍ Other:
Water treatment **between flocks**	❍No	❍ Yes – please specify **products used**:
		❑ Chlorine ❑ Reverse osmosis ❍ Don't know<br>❑ Chlorine dioxide ❑ Sodium Hydrogen Sulfate (e.g., PWT)<br>❑ H. peroxide ❑ Premise disinfectants (e.g., aldehydes)<br>❑ H_2O acidifiers ❑ Ultraviolet<br>❑ Iodine ❑ Other (please specify):______________
Water treatment **during growing period (birds present)**	❍No	❍Yes – please specify products used ***if different from above:***

[1] Self-Evaluation Checklist. National Avian On-Farm Biosecurity Standard. http://www.inspection.gc.ca/english/anima/biosec/aviafrme.pdf.
[2] Downtime: A period of time between flocks, starting with a barn being emptied of birds and ending with the placement of new birds. It allows for the natural reduction in numbers of disease causing micro-organisms within the barn (Safe, Safer, Safest. CFC OFFSP Program).

**Flock ID Code:**	BCSF
**VET ID Code:**	BCSV

17. LIST ALL OF THE RATIONS fed to this flock (BARN LEVEL) during the entire broiler grow-out period.

Attention: These data are critical for our analysis!

Ration #	Ration Name	Start (Age in days)	End (Age in days)	Total Days Fed
1				
2				
3				
4				
5				
6				
7				
NOTE:	THIS TOTAL SHOULD EQUAL THE TOTAL GROWING PERIOD (DAYS) FROM CHICK PLACEMENT TO SAMPLING DAY	⇨	Total # of days:	

18. In the table below complete one line for each ration listed above including non-medicated rations.

- The ration names provided in the table above (Question 17**) MUST correspond** to the ration names used in the table below**.**
- If for one of the above named rations there is **ANY** change in **MEDICATION** then fill in a new line for each change e.g., medicated to non-medicated, change in inclusion rates, change in drug incorporated. You do not need to start a new line if there is a change in ration formulation from a nutrient perspective. A new line is only needed for medication changes.
- Additional space is available over the next page.
- You may use Appendix 2 as a reference to fill in the third column, specify in this table if the antimicrobial is not listed in the appendix.
- Tonnes fed is no longer required; the breed standards will be used to estimate feed consumption.

Attention: These data are critical for our analysis. Please fill in all of the information requested.

**RATION NAME** (From #17) **Please use one line for each antimicrobial per ration and check corresponding PRIMARY reason for each)**	**IS THE FEED MEDICATED?**	**NAME OF ACTIVE INGREDIENTS** (You may use **Appendix 2** for reference)	**GRAMS OF ACTIVE INGREDIENT per Tonne**	**Veterinary prescription available?**	**Primary Reason For Medication Use** ***(PLEASE CHOOSE ONLY ONE PRIMARY REASON)***		
					**Growth promotion**	If Disease **Prevention** ***(CIRCLE all that apply)*** A Yolksacculitis B Septicemia C Musculoskeletal D Respiratory E Necrotic enteritis F Coccidiosis G Other diseases	If Disease **Treatment** ***(CIRCLE all that apply)*** A Yolksacculitis B Septicemia C Musculoskeletal D Respiratory E.Necrotic enteritis F Coccidiosis G Other diseases
	❍Yes ❍No		Grams/tonne	❍Yes ❍No	❍Yes ❍No	A B C D E F G	A B C D E F G
	❍Yes ❍No		Grams/tonne	❍Yes ❍No	❍Yes ❍No	A B C D E F G	A B C D E F G
	❍Yes ❍No		Grams/tonne	❍Yes ❍No	❍Yes ❍No	A B C D E F G	A B C D E F G
	❍Yes ❍No		Grams/tonne	❍Yes ❍No	❍Yes ❍No	A B C D E F G	A B C D E F G
	❍Yes ❍No		Grams/tonne	❍Yes ❍No	❍Yes ❍No	A B C D E F G	A B C D E F G
	❍Yes ❍No		Grams/tonne	❍Yes ❍No	❍Yes ❍No	A B C D E F G	A B C D E F G

FEED MEDICATION, CONTINUED

RATION NAME (From #17) **Please use one line for each antimicrobial per ration and check corresponding PRIMARY reason for each)**	IS THE FEED MEDICATED?	NAME OF ACTIVE INGREDIENTS (You may use **Appendix 2** for reference)	GRAMS OF ACTIVE INGREDIENT per Tonne	Veterinary prescription available?	**Primary** Reason For Medication Use *(PLEASE CHOOSE ONLY ONE PRIMARY REASON)* — Growth promotion	If Disease **Prevention** *(CIRCLE all that apply)*: A Yolksacculitis, B Septicemia, C Musculoskeletal, D Respiratory, E Necrotic enteritis, F Coccidiosis, G Other diseases	If Disease **Treatment** *(CIRCLE all that apply)*: A Yolksacculitis, B Septicemia, C Musculoskeletal, D Respiratory, E.Necrotic enteritis, F Coccidiosis, G Other diseases
	❍Yes ❍No		Grams/tonne	❍Yes ❍No	❍Yes ❍No	A B C D E F G	A B C D E F G
	❍Yes ❍No		Grams/tonne	❍Yes ❍No	❍Yes ❍No	A B C D E F G	A B C D E F G
	❍Yes ❍No		Grams/tonne	❍Yes ❍No	❍Yes ❍No	A B C D E F G	A B C D E F G
	❍Yes ❍No		Grams/tonne	❍Yes ❍No	❍Yes ❍No	A B C D E F G	A B C D E F G
	❍Yes ❍No		Grams/tonne	❍Yes ❍No	❍Yes ❍No	A B C D E F G	A B C D E F G
	❍Yes ❍No		Grams/tonne	❍Yes ❍No	❍Yes ❍No	A B C D E F G	A B C D E F G
	❍Yes ❍No		Grams/tonne	❍Yes ❍No	❍Yes ❍No	A B C D E F G	A B C D E F G
	❍Yes ❍No		Grams/tonne	❍Yes ❍No	❍Yes ❍No	A B C D E F G	A B C D E F G

19. ANTIMICROBIALS VIA DRINKING WATER for the cycle. Please report barn-level medication. Please indicate brand name or the number codes in APPENDIX 2, specify in this table if not listed in the appendix**.**

Check here ❍ if NO antimicrobials added to the drinking water (water medications) during this period.

**Antimicrobial used** Indicate brand name or number codes from Appendix 2	**Total packages used throughout the duration of treatment** Please Indicate units: (e.g., grams/container, Liters/jug, IU/Liter)	**AGE MEDICATED** (Indicate age interval in days; e.g., 24-29 days)	Is the **FLOOR** sampled medicated at least once?	ESTIMATED % of **BARN** medicated at least once	**Prescription provided?**	**Over the counter (OTC) purchase?**	**Primary Reason For Medication Use** ***(PLEASE CHOOSE ONLY ONE PRIMARY REASON)***		
							**Growth promotion**	If Disease **Prevention** (***CIRCLE all that apply***) A Yolksacculitis B Septicemia C Musculoskeletal D Respiratory E Necrotic enteritis F Coccidiosis G Other diseases	If Disease **Treatment** (***CIRCLE all that apply***) A Yolksacculitis B Septicemia C Musculoskeletal D Respiratory E Necrotic enteritis F Coccidiosis G Other diseases
	)	___ - ___ days	❍ Yes ❍ No	%	❒	❒	❍Yes ❍No	A B C D E F G	A B C D E F G
		___ - ___ days	❍ Yes ❍ No	%	❒	❒	❍Yes ❍No	A B C D E F G	A B C D E F G
		___ - ___ days	❍ Yes ❍ No	%	❒	❒	❍Yes ❍No	A B C D E F G	A B C D E F G
		___ - ___ days	❍ Yes ❍ No	%	❒	❒	❍Yes ❍No	A B C D E F G	A B C D E F G
		___ - ___ days	❍ Yes ❍ No	%	❒	❒	❍Yes ❍No	A B C D E F G	A B C D E F G

20. FLOCK HEALTH STATUS (***PLEASE check all applicable based on overall clinical assessment of the flock, health records or by asking the producer; these are critical for our analysis. If no response was provided for a specific disease, we will assume this to be LIKELY NEGATIVE.***)

Check here ❍ if there was no disease syndrome noted during the grow-out period.

Infectious diseases/syndromes	FLOCK DISEASE STATUS				DIAGNOSIS WAS BASED ON (Check all that apply)			WERE ANTIMICROBIALS USED TO CONTROL OR TREAT THIS CONDITION?		
	Confirmed Negative	Likely Negative	Likely Positive	Confirmed Positive	Clinical signs	Post-mortem	Laboratory testing	YES	NO	DON'T KNOW
A. Yolksacculitis	❍	❍	❍	❍	❒	❒	❒	❍	❍	❍
B. Septicemia	❍	❍	❍	❍	❒	❒	❒	❍	❍	❍
C. Necrotic Enteritis	❍	❍	❍	❍	❒	❒	❒	❍	❍	❍
D. Staphylococcal/ tenosynovitis	❍	❍	❍	❍	❒	❒	❒	❍	❍	❍
E. Vertebral Osteomyelitis/ Spondylitis	❍	❍	❍	❍	❒	❒	❒	❍	❍	❍
F. Infectious Bursal Disease (IBD)	❍	❍	❍	❍	❒	❒	❒	❍	❍	❍
G. Inclusion Body Hepatitis (IBH)	❍	❍	❍	❍	❒	❒	❒	❍	❍	❍
H. Infectious Bronchitis (IBV)	❍	❍	❍	❍	❒	❒	❒	❍	❍	❍
I. Chicken Anemia Virus (CAV)	❍	❍	❍	❍	❒	❒	❒	❍	❍	❍
J. Reovirus	❍	❍	❍	❍	❒	❒	❒	❍	❍	❍
K. Paratyphoid *Salmonella*	❍	❍	❍	❍	❒	❒	❒	❍	❍	❍
L. Coccidiosis	❍	❍	❍	❍	❒	❒	❒	❍	❍	❍
M.Airsacculitis	❍	❍	❍	❍	❒	❒	❒	❍	❍	❍
N. Other infections: please specify	❍	❍	❍	❍	❒	❒	❒	❍	❍	❍

21. VACCINES AND OTHER PRODUCTS ADMINISTERED TO FLOCK AFTER CHICK PLACEMENT (CHECK ALL APPLICABLE AND **INDICATE AGE** OF FLOCK AT ADMINSTRATION)

Check here ❍ if NO vaccines/bacterins were administered DURING GROW-OUT PERIOD.

Agent	Trade names/brand, manufacturer	Days administered
Coccidiosis vaccines	❑ Coccivac-B52 (Merck AH)	days
	❑ Coccivac-D2 (Merck AH)	days
	❑ Hatchpak Cocci III (Merial-BI	days
	❑ Immucox for chickens I (Ceva AH)	days
	❑ Immucox for chickens II (Ceva AH)	days
	❑ Others, *please specify:* ___________________________	days
Bronchitis vaccines	❑ Bronchitis Vaccine, Mass. (Zoetis)	days
	❑ Bronchitis Vaccine, Mass. freeze-dried (Merial-BI)	days
	❑ IB Vac H, Mass. Holland Strain (Merck AH)	days
	❑ Mildvac Ma5, Mass. (Merck AH)	days
	❑ Bronchitis Vaccine, Mass + Conn (Zoetis)	days
	❑ Others *please specify:* _____________________________	days
Infectious Bursal Disease	❑ Avipro Vibursa CE (LAHI)	days
	❑ Bursal Disease S-706 (Merial-BI)	days
	❑ Bursa Blen M (Merial-BI)	days
	❑ Bursavac (Merck AH)	days
	❑ Bursimune (Ceva AH)	days
	❑ Bursine 2 (Zoetis)	days
	❑ Clonevac D78 (Merck AH)	days
	❑ Poulvac Bursaplex (Zoetis)	days
	❑ SVS 510 (Merial-BI)	days
	❑ Univax BD (Merck AH)	days
	❑ Univax Plus (Merck AH)	days
	❑ Others *please specify:*	days
Newcastle Disease	❑ *Please specify:*	days
Reovirus	❑ Enterovax (Merck AH)	days
	❑ Others *please specify, e.g., autogenous:*	days
Bacterial agents	❑ *Salmonella*, Avipro MeganVAC1 (LAHI)	days
	❑ *Salmonella,* Poulvac ST (Zoetis)	days
	❑ *Salmonella*, Salmune (Ceva AH)	days
	❑ *E. coli*, Poulvac *E. coli* (Zoetis)	days
	❑ Others, *please specify* :	days
Other bacterial or viral agents	❑ *Please specify:*	days
	❑ *Please specify:*	days
**Non-antimicrobial additives or animal health products**		**Duration of treatment**
	❑ Acidifiers: *please specify* (e.g., Gallinat+, Gallinat600)	days
	❑ Prebiotics: *please specify* (e.g., Actigen, Citristim):	days
	❑ Probiotics: *please specify* (e.g., Floractin):	days
	❑ Others, *please specify*:	days

Appendix 1. Premise disinfectant list for poultry (Source: Compendium of Vet. Products Online, Accessed: November, 2017)

Active ingredient	Brand name
**Chlorhexidine**	Hibitane® Disinfectant (Zoetis)
**Chlorine/sulfate**	Virkon® Disinfectant/Cleaner P.W.S. Virucide (Vétoquinol)<br>Virkon® Tablets (Vétoquinol)
**Hydrogen peroxides**	Cid 2000 (Merial)<br>HemaPeroxy Liquid Disinfectant (AVL)<br>Hyperox (Vétoquinol)
**Iodine complex**	Barn-Storm Iodine Cleaner Sanitizer (Ostrem)<br>Biodine (Dominion)<br>Premise Disinfectant (West Penetone)<br>Wescodyne* (West Penetone)
**Phenol**	1-Stroke Environ® (Steris)<br>LpH ag® (Steris)<br>Multi-Phenolic Disinfectant (Bio Agri Mix)<br>Tek-trol (Pro Ag)
**Quaternary ammonium (quat)**	Amazing! Nok Out™ Odor Eliminator (OdorTECH)<br>BioSentry® 904 Disinfectant (Hacco)<br>BioSentry® 904 Disinfectant (Vétoquinol)<br>BioSentry® BioQuat™ 20 Disinfectant (Hacco)<br>BioSentry® BioQuat™ 20 Disinfectant (Vétoquinol)<br>Clinicide (Bimeda-MTC)<br>Coverage 256® (Steris)<br>Gluquat (West Penetone)<br>PF 300 (DuBois)<br>Proquat™ (Engage)<br>Viropex 1 (Agrisan)
**Various ingredient**	Aseptol 2000® (SEC Repro)<br>Amazing! Knok-out (Odortek)<br>BioSentry® Acid-A-Foam™ (Hacco)<br>BioSentry® Acid-A-Foam™ (Vétoquinol)<br>BioSentry® Liquid Tray & Egg Wash (Hacco)<br>BioSentry® Liquid Tray & Egg Wash (Vétoquinol)<br>Fumalyse II (Bio Agri Mix)<br>Premicide 277 (Agrisan)<br>Profilm® (Hacco)<br>Profilm® (Vétoquinol)<br>Spectragen (SEC Repro)<br>Synergize (Pro-Ag)<br>Virocid® (Merial)
**Benzyl ammonium chloride**	Bioxy Enviro (UKAL Canada)

Appendix 2. Veterinary Drug Listing, Canada (Source: Compendium of Vet. Products Online, Accessed Nov. 24, 2017)

A. In-feed medications

Codes	*Brand name*	*Active ingredient*
**F1**	BMD® 110 G	Bacitracin
**F2**	Bacitracin MD (BioAgri-mix)	
**F3**	Flavomycin® 4 (Huvepharma AD)	Bambermycin
**F4**	Aureomycin® 220 G (Zoetis)	Chlortetracycline
**F5**	Chlor 100 Medicated Premix (BioAgri Mix)	
**F6**	Gallimycin® 50 (BioAgri-Mix)	Erythromycin
**F7**	Oxysol-220	Oxytetracycline
**F8**	Oxysol-440	
**F9**	Oxytetracycline 50 Premix (BioAgri Mix)	
**F10**	Oxytetracycline 100 Premix (BioAgri Mix)	
**F11**	Oxytetracycline 200 Premix (BioAgri Mix)	
**F12**	Terramycin® 50 Premix (Phibro)	
**F13**	Terramycin® 100 Premix (Phibro)	
**F14**	Terramycin® 200 Premix (Phibro)	
**F15**	Penicillin G Procaine 110 (BioAgri Mix)	Penicillin G Procaine
**F16**	Tylan® 10 (Elanco)	Tylosin phosphate
**F17**	Tylan® 40 (Elanco)	
**F18**	Tylan® 100 (Elanco)	
**F19**	Tylosin 40 (BioAgri Mix)	
**F20**	Stafac® 22 (Phibro)	Virginiamycin
**F21**	Stafac® 44 (Phibro)	
**F22**	Stafac® 500 (Phibro)	
**F23**	Virginiamycin 44 Premix (BioAgri Mix)	
**F24**	**Uniprim**	**Trimethoprim-sulfadiazine**
**F25**	**Surmax**	**Avilamycin**
Anticoccidials		
**F26**	Amprol® 25% Feed Mix (Huvepharma AD)	Amprolium
**F27**	Coyden® 25% (Huvepharma AD)	Clopidol
**F28**	Deccox® 6% Premix (Zoetis)	Decoquinate
**F29**	Clinacox® 0.5% Premix (Elanco)	Diclazuril
**F30**	Avatec® 20 Medicated Premix (Zoetis)	Lasalocid
**F31**	Bovatec 20 Medicated Premix (Zoetis)	
**F32**	Cygro® 1% Premix (Zoetis)	Maduramicin
**F33**	Coban® Premix (Elanco)	Monensin
**F34**	Monensin Premix (BioAgri Mix)	
**F35**	Rumensin® Premix (Elanco)	
**F36**	Monteban® 100 Elanco	Narasin
**F37**	Maxiban®	Narasin-Nicarbazin
**F38**	Nicarb® (Huvepharma AD)	Nicarbazin
**F39**	Robenz® Medicated Feed (Zoetis)	Robenidine
**F40**	Coxistac® 6% Premix (Phibro)	Salinomycin
**F41**	Coxistac® 12% Premix (Phibro)	
**F42**	Sacox® 120 (Huvepharma AD)	
**F43**	Salinomycin 60 Premix (BioAgri Mix)	
**F44**	Posistac® 6% (Phibro)	
**F45**	Zoamix® (Huvepharma AD)	Zoalene
Other antimicrobials/anticoccidials (new/not listed)		
**F46**		
**F47**		
**F48**		
**F49**		
**F50**		

B. Water medication

Codes	Brand name	Active ingredient
W1	Amoxicillin SP (BioAgri Mix)	Amoxicillin
W2	Paracillin® SP (Merck AH)	
W3	Apralan® (Elanco)	Apramycin sulfate
W4	Lincomix ®Soluble Powder (Zoetis)	Lincomycin
W5	Lincomycin Soluble Powder (BioAgri Mix)	
W6	LinxMed-SP (Bimeda-MTC)	
W7	Lincomycin Spectinomycin 100 Soluble Powder (BioAgri Mix)	Lincomycin+Spectinomycin
W8	Linco-Spectin® 100 Soluble Powder (Zoetis)	
W9	Neomed 325 (Medprodex)	Neomycin
W10	Neomycin 325 (Vetoquinol)	
W11	Neomycin SP (AVL)	
W12	NeoOxytet SP (AVL)	Neomycin+oxytet.
W13	Neotet Soluble Concentrate (Dominion)	
W14	Neo-Chlor® (Vetoquinol)	Neomycin+tetracycline
W15	Neo-Tetramed (Medprodex)	
W16	Oxy 250 (Medprodex)	Oxytetracycline
W17	Oxy 1000 (Jaapharm)	
W18	Oxysol 62.5 (Vetoquinol)	
W19	Oxy Tetra-A (Dominion)	
W20	Oxy Tetra Forte (Dominion)	
W21	Oxytetracycline HCl Soluble Powder 1,000 (BioAgri Mix)	
W22	Booster P S Conc. (Jaapharm)	Penicillin+Streptomycin+ Vitamin(s)
W23	Super Booster™ (Vetequinol)	
W24	Vibiomed Booster (Medprodex)	
W25	Penicillin G Potassium USP Soluble, 500 M IU (BioAgri Mix)	Penicillin G potassium
W26	Penicillin G Potassium USP Soluble, 15,000 M IU (BioAgri Mix)	
W27	Pot-Pen™, 500 M IU (Vetoquinol)	
W28	Pot-Pen™, 15,000 M IU (Vetoquinol)	
W29	Sodium Sulfamethazine 12.5% Solution (Dominion)	Sulfamethazine
W31	Sodium Sulfamethazine 25% Solution (Dominion)	
W32	Sodium Sulfamethazine 25% Solution (PVL)	
W33	Sulfa 25% Solution (Bimeda-MTC)	
W34	Sulfamethazine 25 Solution (AVL)	
W35	Onycin 250 (Vetoquinol)	Tetracycline
W36	Onycin 1000 (Vetoquinol)	
W37	Tetra 55 (Jaapharm)	
W38	Tetra 250 (Jaapharm)	
W39	Tetra 1000 (Jaapharm)	
W40	Tetra 1000 (Dominion)	
W41	Tetra 250 (Vetoquinol)	
W42	Tetracycline Hydrochloride (Dominion)	
W43	Tetracycline Hydrochloride (PVL)	
W44	Tetramed 250 (Medprodex)	
W45	Tetramed 1000 (Medprodex)	
W46	Tylan® Soluble (Elanco)	Tylosin tartrate
W47	Tylosin Soluble Powder (BioAgri Mix)	
Anticoccidial-water medication and other antimicrobials/anticoccidials (new, not listed)		
W48	Amprol® 9.6% Solution (Huvepharmad AD)	Amprolium
W49	Ampromed (Bimeda MTC)	
W50	Quinnoxine-S (Vetoquinol)	Pyrimethamine-Sulfaquinoxaline
W51	Sulfaquinoxaline Concentrate 19.2% (AVL)	Sulfaquinoxaline
W52	Sulfaquinoxaline 19.2% Liquid Concentrate (Dominion)	Sulfaquinoxaline
W53	Baytril	Enrofloxacin
W54		
W55		
