# Supplementary material for: Antimicrobial Use Indices—The Value of Reporting Antimicrobial Use in Multiple Ways Using Data From Canadian Broiler Chicken and Turkey Farms
Source: Front Vet Sci. 2020 Oct 19;7:567872. doi: 10.3389/fvets.2020.567872 (PMC7604299; doi:10.3389/fvets.2020.567872)

Supplementary Materials II

A. BROILERS

Annex 1 | Summary of the antimicrobial use data reported by frequency, weight-based and dose-based indicators, by antimicrobial class at the flock-level in broiler chickens, 2013 to 2019.

	Mean of flocks (Standard error of the mean)			
**Antimicrobial class**	**Treated flocks n (%)**	**mg/PCU**	**nDDDvetCA/1,000 broiler-chicken days at risk**	**nDDDvetCA/PCU**
Fluoroquinolones	3 (0.3%)	0.4 (0.1)	2 (0.3)	0.1 (0.01)
Third generation cephalosporins	39 (4%)	0.1 (0.01)	1 (0.1)	0.05 (0.004)
Aminoglycosides	65 (7%)	13 (4)	19 (5)	4 (0.2)
Lincosamides and aminocyclitols	178 (19%)	1 (0.4)	7 (3)	0.4 (0.1)
Macrolides	91 (10%)	43 (3)	48 (3)	3 (0.1)
Penicillins	139 (15%)	77 (7)	246 (17)	7 (1)
Streptogramins	192 (21%)	48 (2)	481 (20)	2 (1)
Trimethoprim and sulfonamides	106 (11%)	156 (12)	594 (50)	12 (2)
Bacitracin	509 (54%)	144 (3)	406 (8)	3 (0.3)
Tetracycline	19 (2%)	190 (60)	275 (83)	60 (4)
Orthosomycins	213 (23%)	31 (1)	307 (12)	1 (0.4)

Annex 2 | Distribution of quantities of antimicrobials reported to be used, by antimicrobial classes in milligrams (mg) per population correction unit (PCU) in treated broiler chicken flocks, 2013 to 2019.

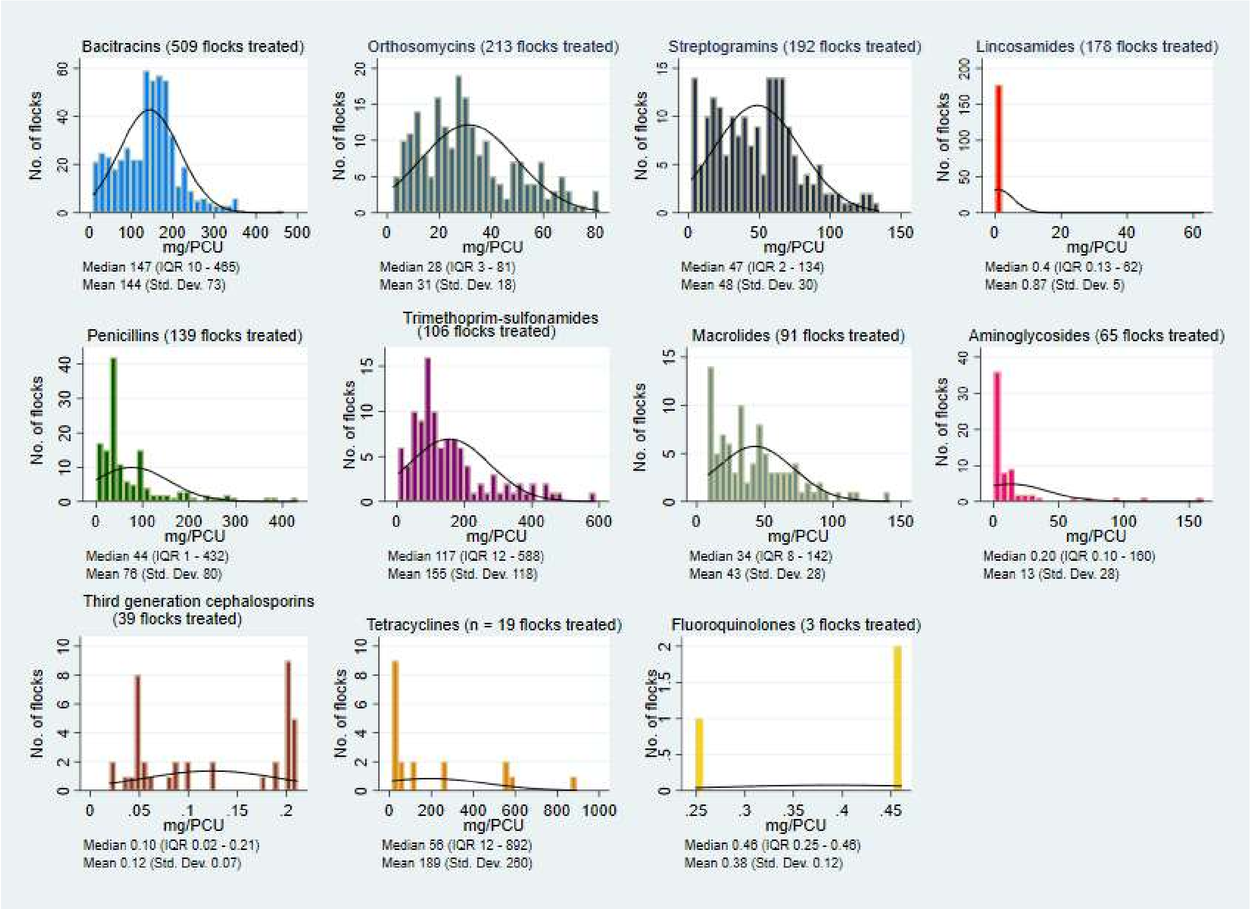

Antimicrobial classes were organized from high to low frequency of use; we caution our readers that the scale (both axes) varied considerably depending on the class and antimicrobial active ingredients. Please refer to Table 3 in the main documen.

Annex 3 | Pairwise correlation between antimicrobial use indicators in broiler chicken and turkey flocks. Data were transformed to the log scale for better visualization.

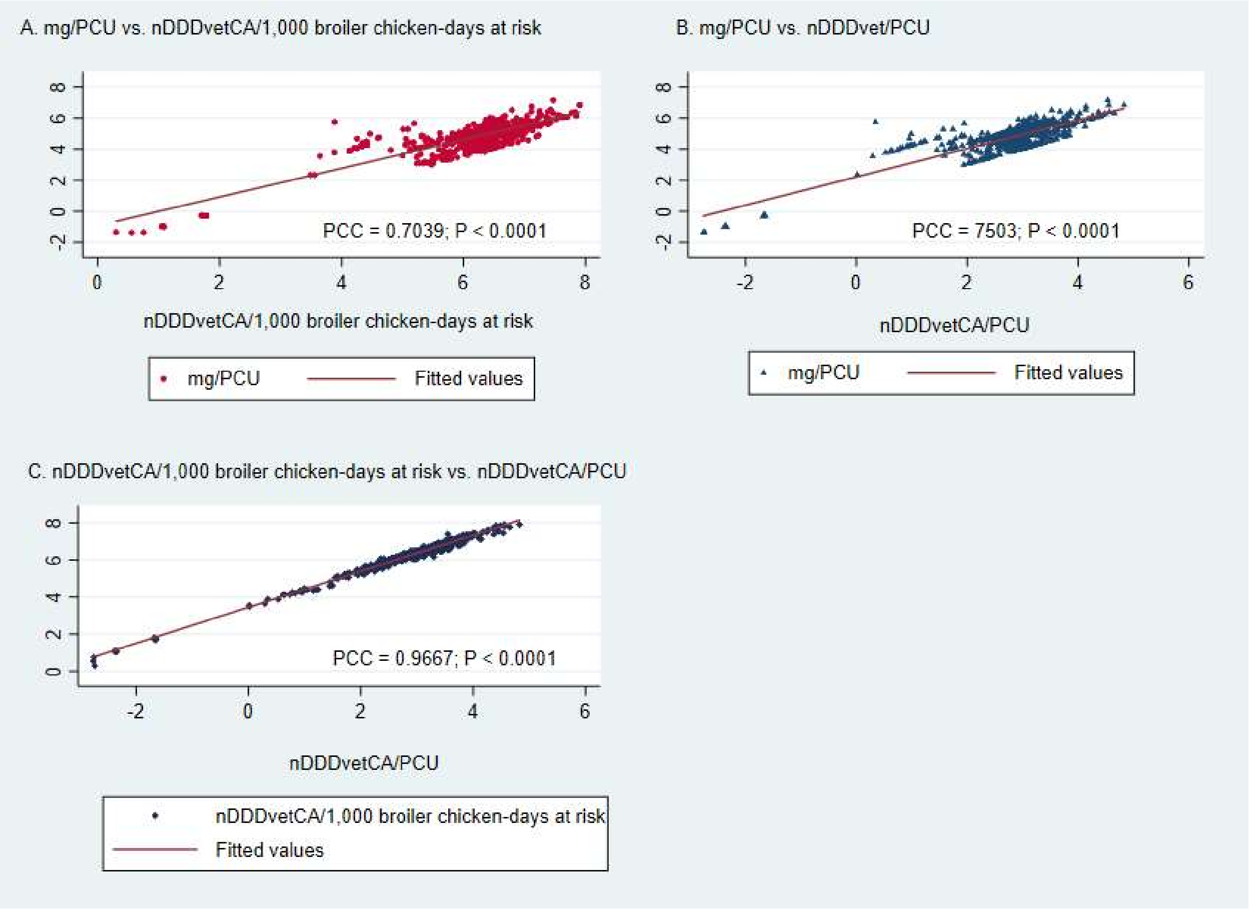

Annex 4 | Quantity of antimicrobials in milligrams adjusted for population correction unit by routes of administration in broiler chicken flocks, 2013 to 2019.

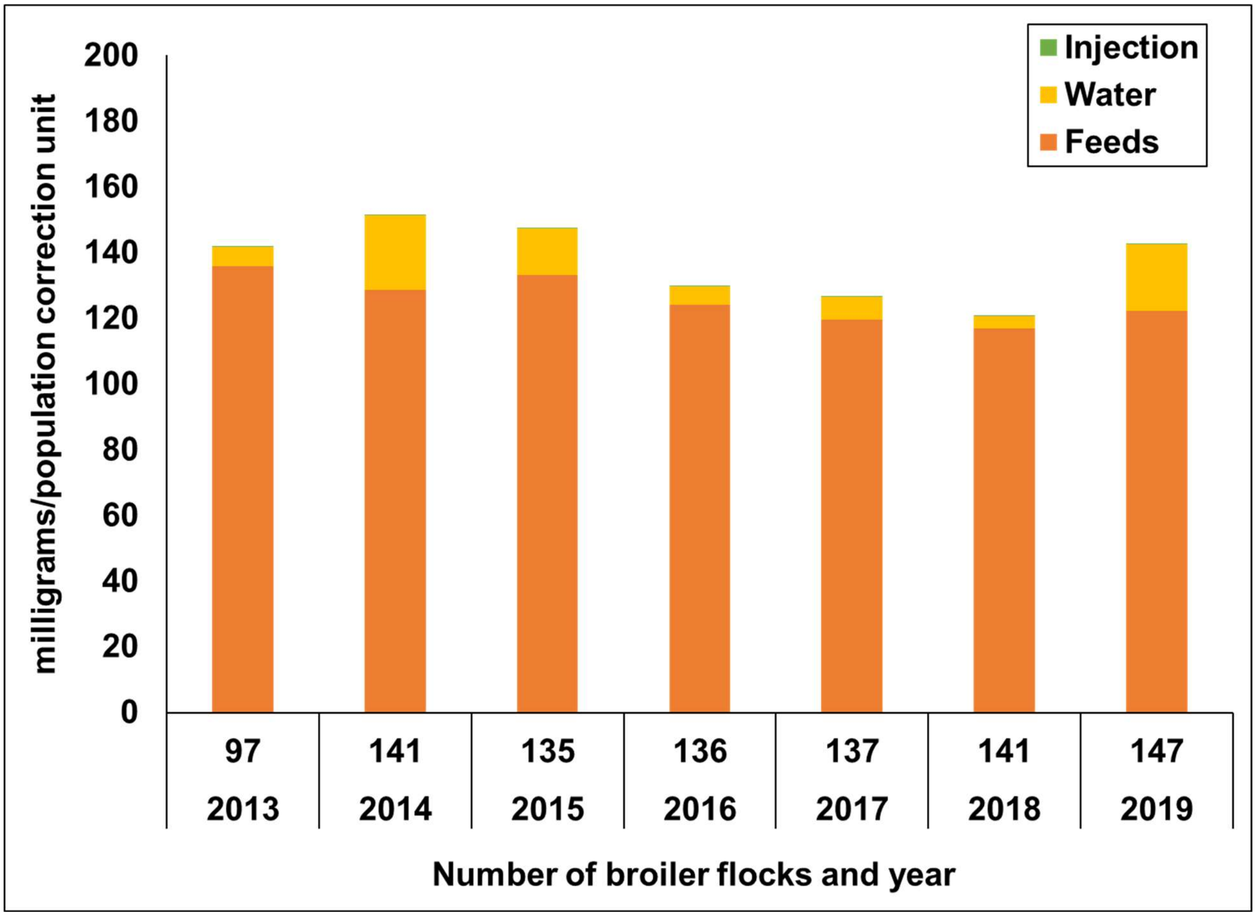

B. Turkeys

Annex 5 | Summary of the antimicrobial use data reported by frequency, weight-based and dose-based indicators, by antimicrobial class at the flock-level in turkeys, 2013 to 2019.

Antimicrobial class	Treated flocks, n (%)	Mean of flocks (Standard error of the mean)		
		**mg/PCU**	**nDDDvetCA/1,000 turkey-days at risk**	**nDDDvetCA/PCU**
Fluoroquinolones	4 (1%)	1 (0.3)	3 (1)	0.2 (0.05)
Third generation cephalosporins	1 (1%)	< 0.1	< 0.1	< 0.1
Aminoglycosides	195 (46%)	1 (0.3)	0.5 (0.2)	0.05 (0.02)
Macrolides	9 (2%)	44 (10)	17 (4)	2 (0.4)
Penicillin	47 (11%)	24 (7)	17 (3)	2 (0.3)
Streptogramins	130 (30%)	33 (2)	131 (5)	12 (1)
Trimethoprim and sulfonamides	22 (5%)	109 (21)	173 (31)	17 (3)
Bacitracins	181 (42%)	96 (5)	103 (4)	9 (0.5)
Tetracyclines	26 (6%)	62 (16)	36 (9)	4 (1)
Orthosomycins	10 (2%)	19 (5)	69 (13)	6 (2)

Annex 6 | Distribution of quantities of antimicrobials reported to by used, by antimicrobial classes in milligrams (mg) per population correction unit (PCU) in treated turkey flocks, 2013 to 2019.

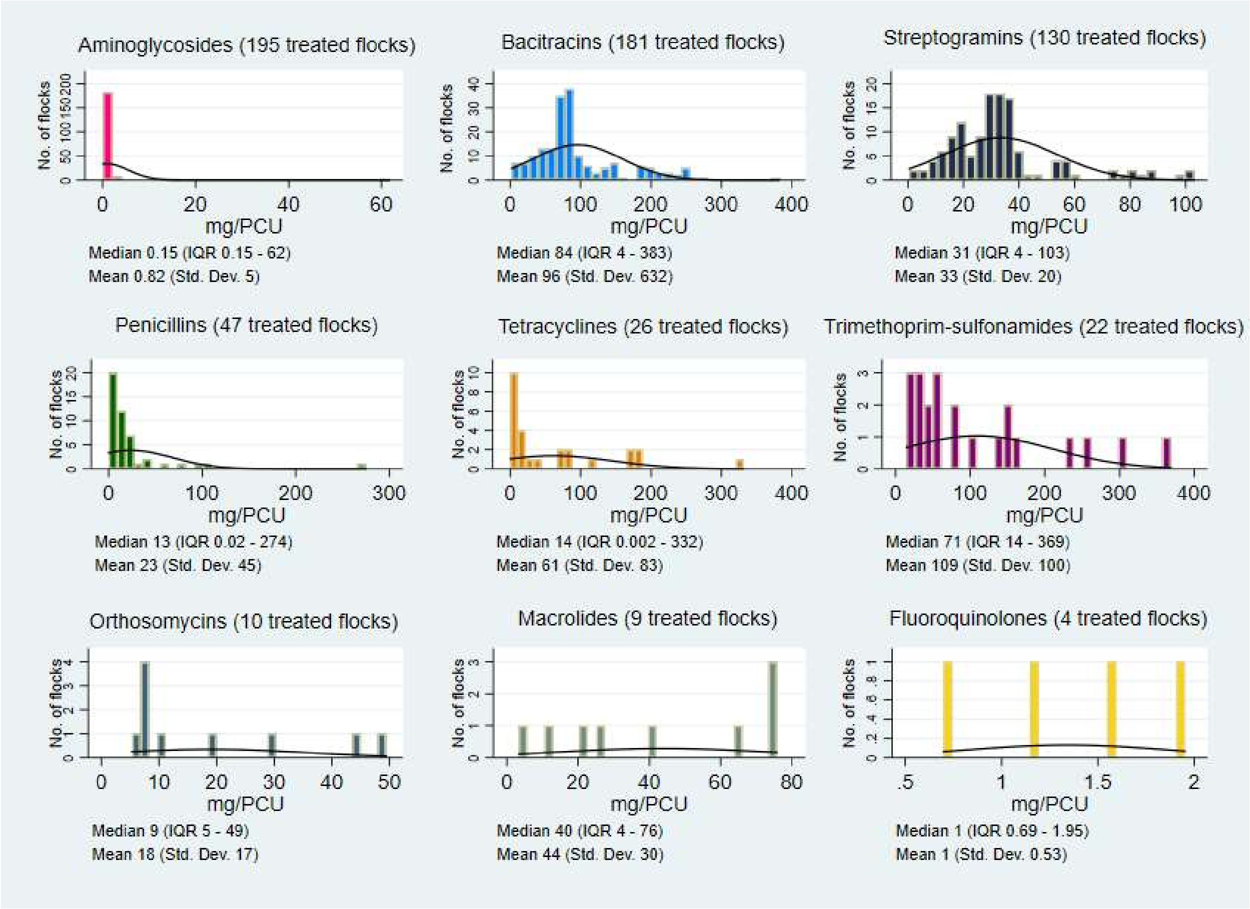

Antimicrobial classes were organized from high to low frequency of use; we caution our readers that the scale (both axes) varied considerably depending on the class and antimicrobial active ingredient. Please refer to Table 5 in the main document.

Annex 7 | Pairwise correlation between antimicrobial use indicators in turkey flocks. *Data were transformed to the log scale for better visualization*

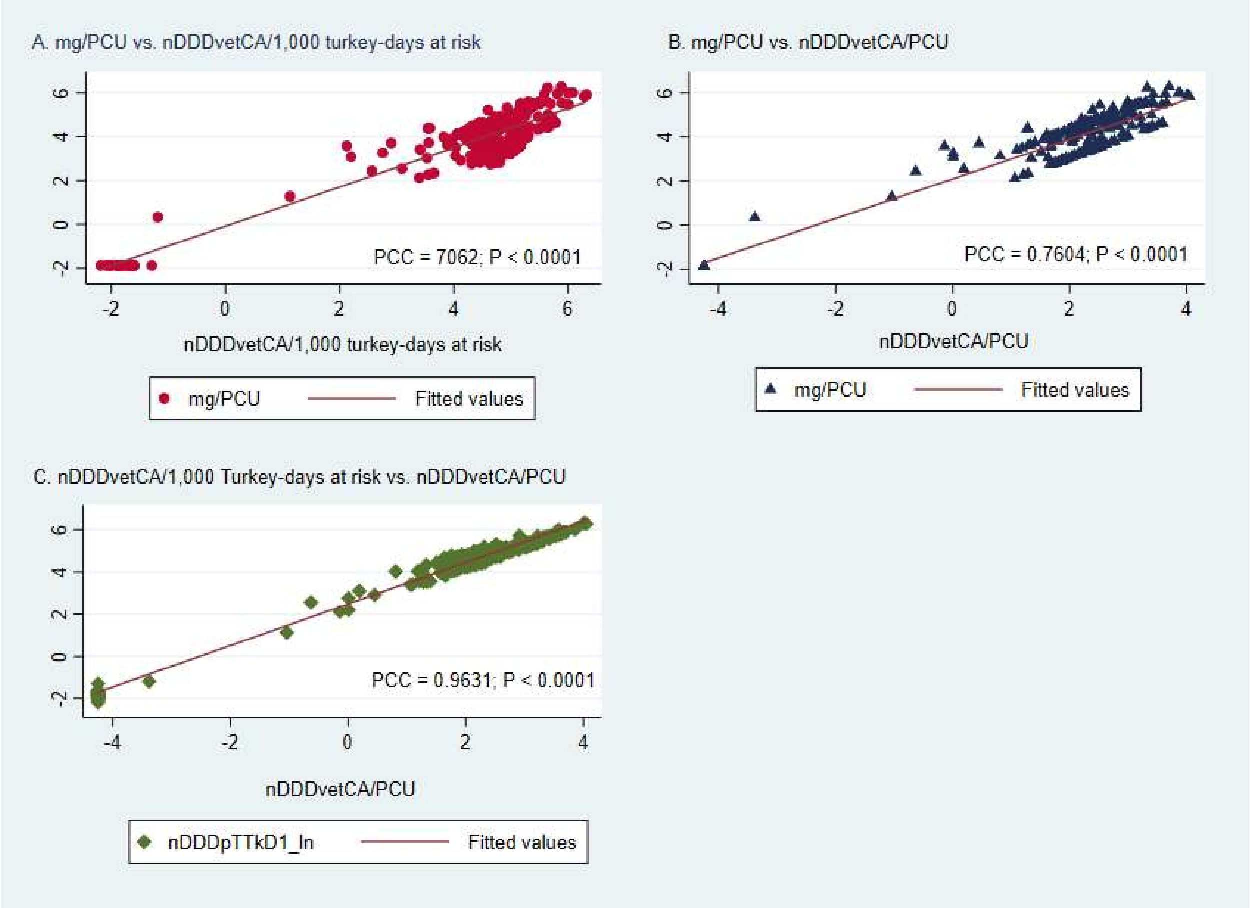

Annex 8 | Quantity of antimicrobials in milligrams adjusted for population correction unit by routes of administration in turkey flocks, 2013 to 2019.

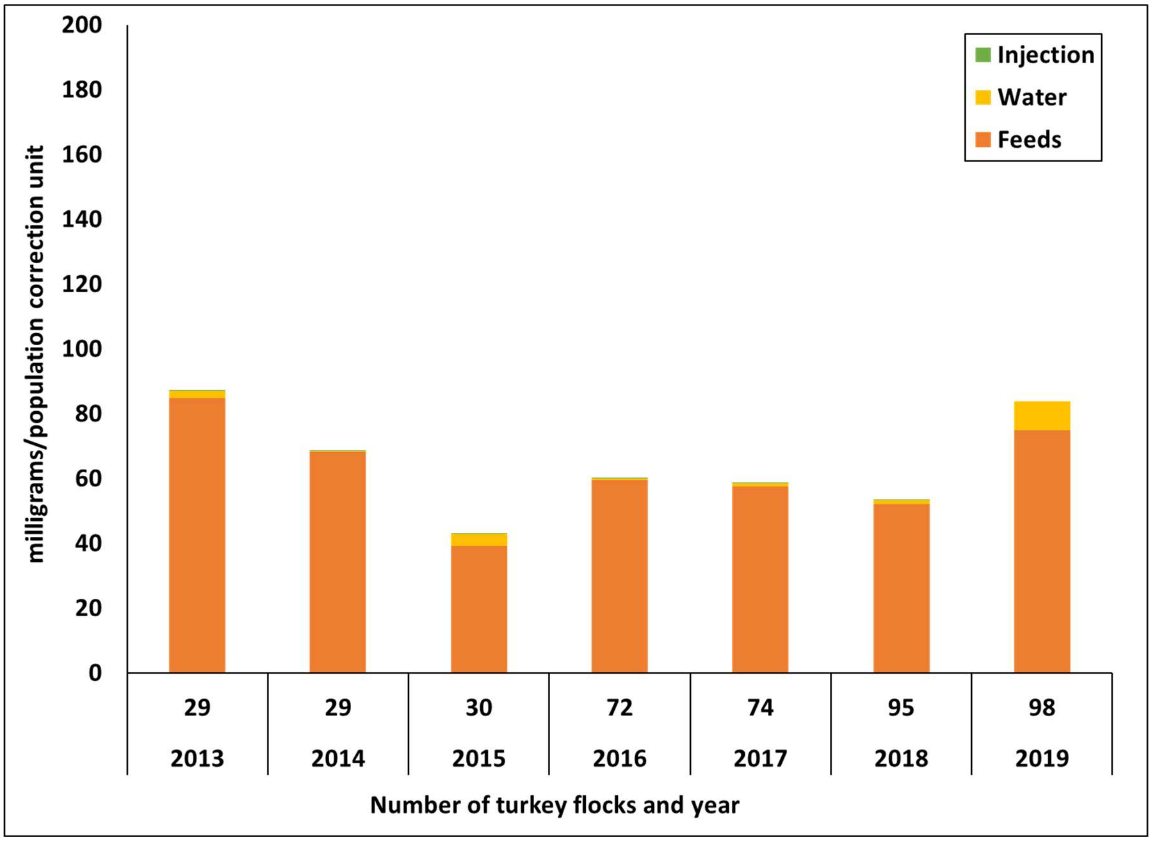

Supplement: Supplementary Material 3 — Other findings. Other antimicrobial use descriptive statistics and correlation matrices. [file Data_Sheet_3.pdf]
